# Improved Performance of Organic Thermoelectric Generators Through Interfacial Energetics

**DOI:** 10.1002/advs.202206954

**Published:** 2023-05-03

**Authors:** I. Petsagkourakis, S. Riera‐Galindo, T.‐P. Ruoko, X. Strakosas, E. Pavlopoulou, X. Liu, S. Braun, R. Kroon, N. Kim, S. Lienemann, V. Gueskine, G. Hadziioannou, M. Berggren, M. Fahlman, S. Fabiano, K. Tybrandt, X. Crispin

**Affiliations:** ^1^ Laboratory of Organic Electronics Department of Science and Technology (ITN) Linköping University SE‐601 74 Norrköping Sweden; ^2^ Bordeaux INP CNRS Univ. Bordeaux LCPO F‐33600 UMR 5629 Pessac France; ^3^ Wallenberg Wood Science Center Linköping University 602 23 Norrköping Sweden; ^4^ Institute of Electronic Structure and Laser, Foundation for Research and Technology 71110 Heraklion Crete Greece; ^5^ Present address: RISE Research Institutes of Sweden Digital Systems Smart Hardware Bio‐ and Organic Electronics SE‐602 21 Bredgatan 33 Norrköping Sweden; ^6^ Present address: Institut de Ciencia de Materials de Barcelona (ICMAB‐CSIC) Campus de la UAB 08193 Carrer dels Til lers, s/n, Bellaterra Barcelona Spain; ^7^ Present address: Smart Photonic Materials Faculty of Engineering and Natural Sciences Tampere University 33720 Tampere Finland

**Keywords:** interface, organic electronics, organic thermoelectric generator, polymer thermoelectrics, Seebeck coefficient, thermoelectric

## Abstract

The interfacial energetics are known to play a crucial role in organic diodes, transistors, and sensors. Designing the metal‐organic interface has been a tool to optimize the performance of organic (opto)electronic devices, but this is not reported for organic thermoelectrics. In this work, it is demonstrated that the electrical power of organic thermoelectric generators (OTEGs) is also strongly dependent on the metal‐organic interfacial energetics. Without changing the thermoelectric figure of merit (*ZT*) of polythiophene‐based conducting polymers, the generated power of an OTEG can vary by three orders of magnitude simply by tuning the work function of the metal contact to reach above 1000 µW cm^−2^. The effective Seebeck coefficient (*S*_eff_) of a metal/polymer/metal single leg OTEG includes an interfacial contribution (*V*_inter_/Δ*T*) in addition to the intrinsic bulk Seebeck coefficient of the polythiophenes, such that *S*_eff_ = S + *V*_inter_/Δ*T* varies from 22.7 µV K^−1^ [9.4 µV K^−1^] with Al to 50.5 µV K^−1^ [26.3 µV K^−1^] with Pt for poly(3,4‐ethylenedioxythiophene):*p*‐toluenesulfonate [poly(3,4‐ethylenedioxythiophene):poly(4‐styrenesulfonate)]. Spectroscopic techniques are used to reveal a redox interfacial reaction affecting locally the doping level of the polymer at the vicinity of the metal‐organic interface and conclude that the energetics at the metal‐polymer interface provides a new strategy to enhance the performance of OTEGs.

## Graphical abstract

For the same conducting polymer layer, the power generated by a lateral organic thermoelectric generator (OTEG) can be tuned by three orders of magnitude through the energetics at the metal–polymer interface reaching above 1000 µW cm^−2^. This novel approach towards improving the performance of OTEGs brings us one step closer towards efficient and printed OTEGs.

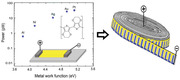

## 1 Introduction

Thermoelectric devices convert heat flux into electricity, and vice versa. Over the last decade, organic (semi)conductors have attracted some attention for thermoelectric applications thanks to a series of compelling and unique properties, such as simple processing and manufacturing, mechanical flexibility, high abundance with respect to their atomic elements, as well as electric‐to‐thermal conductivity ratios (*σ*/*κ)* similar to those of conventional inorganic alloys operating at low temperatures (<200 °C). Various strategies have emerged to increase the Seebeck coefficient (*S*), power factor (*σ S^2^*), and figure of merit *ZT* (= *σ* *S* ^2^ T/*κ*) of thermoelectric polymers.^[^ ^1^, ^2^, ^3^, ^4^ ^]^ Today the best‐performing organic thermoelectric material comprises positively charged poly(3,4‐ethylenedioxythiophene) (PEDOT) chains that are charge‐compensated with anionic counterions X (PEDOT:X) which at an optimum oxidation (doping) level yields *ZT* values in the range 0.25–0.4 at room temperature.^[^ ^5^, ^6^ ^]^ Most of the PEDOT thin films are obtained by the oxidative polymerization of the 3,4‐ethylenedioxythiophene (EDOT) monomer with an oxidizing agent. The strength of the oxidative agent governs the actual doping level of the polymer film. Typically, the oxidation level is ≈30% and can be reduced afterwards by additional post‐treatment. There is a well‐established correlation between the oxidation level, the Seebeck coefficient and the electrical conductivity.^[^ ^5^ ^]^ Although the device geometry has been found to be crucial for measuring the Seebeck coefficient of the materials accurately,^[^ ^7^ ^]^ the reported values without these errors for pristine PEDOT films (without post‐treatment to increase the Seebeck coefficient) still vary from 15 up to 45 µV K^−1^ for PEDOT thin films.^[^ ^1^, ^6^, ^8^, ^9^, ^10^, ^11^, ^12^ ^]^ Those differences found in the literature are not clarified but have a significant impact on the thermoelectric power factor and the generated power density. We have not found any systematic study of the potential impact of the metal contact on the apparent Seebeck coefficient of the PEDOT films.

For an ideal generator, the maximum output power is found for a load resistance that equals the internal resistance (*R*_dev_) of the organic thermoelectric generator (OTEG), and is expressed by:

(1) $$P_{\max}=V_{\mathrm{oc}}^{2}/4R_{\mathrm{dev}}$$

(2) $$V_{\mathrm{oc}}=S\Delta T$$

where *V*_oc_ is the open‐circuit voltage (thermovoltage). In a simplistic model, one leg of a thermoelectric generator is composed of two metal contacts and the thermoelectric material; and the internal resistance does not only come from the bulk of the thermoelectric material but also from its interface with the two metal contacts. In inorganic thermoelectric generators, it is well established that the contact resistance between the metal contact and the thermoelectric material limits the efficiency and power output performance.^[^ ^13^, ^14^ ^]^ Also, the energetics at the metal‐organic semiconductor interface is recognized to play a major role in determining the performance of organic (opto)electronic devices, such as organic transistors and solar cells,^[^ ^15^ ^]^ but a similar dependence has never been identified or even investigated for OTEGs.

Here, we report a systematic study on the impact of the metal‐organic semiconductor interface energetics on the thermoelectric performance of p‐type polymer‐based OTEGs. We observe that while the Seebeck coefficient of PEDOT films does not vary with the work function of the metal contact in inert and dry atmosphere, the thermo‐induced voltage is systematically larger with metals of high work function when measured in humid air. This striking effect indicates that there is redox reaction leading to an interfacial contribution to the thermo‐induced voltage. Hence the measured voltage divided by the temperature gradient provides an apparent Seebeck coefficient related to the device, rather than an intrinsic Seebeck coefficient related to the thermoelectric material. On top of this effect on the Seebeck voltage, the resistance of the device and the overall electrical power density are also strongly contact dependent. Infrared spectroscopy allows us to identify an interfacial redox reaction occurring between PEDOT and the metal contact,^[^ ^16^ ^]^ which modifies the oxidation level of the polymer at the polymer‐metal interface. Learning from these new insights, we tune the interfacial energetics to optimize the electrical power output of OTEGs over three orders of magnitude. We anticipate that our findings of interfacial energetics‐thermopower dependence will have a similar impact on the field of organic thermoelectrics as it has had in the context of organic field‐effect transistors^[^ ^17^, ^18^ ^]^ and organic solar cells.^[^ ^19^ ^]^

## 2 Results

### 2.1 Single Leg Thermoelectric Generators

PEDOT:Poly(4‐styrenesulfonate) (PEDOT:PSS) and PEDOT:*p*‐Toluenesulfonate (PEDOT:Tos) films (≈100 nm thick, 2 mm in width, 20 mm in length) were deposited on glass substrates pre‐patterned with metal contacts (≈100 nm thick). While exposed to ambient air (40% Relative Humidity RH, 20 °C), a temperature gradient Δ*T* is applied between the two electrodes and the open circuit voltage *V*_oc_ between those two electrodes is measured to estimate the Seebeck coefficient *S* = *V*_oc_⁄Δ*T* (Figure S1.1, Supporting Information). The linewidth and inter‐distance separation of the metal electrodes were chosen such to minimize the error associated with the estimation of the Seebeck coefficient (Figure S1.2, Supporting Information), while the samples were stable in air for more than ten days (Figure S1.4, Supporting Information).^[^ ^5^, ^7^ ^]^ Five different metals were explored, Al, Ni, Ag, Au, and Pt, covering a range of work function values from 3.7 to 5.2 eV, as measured by ultraviolet photoelectron spectroscopy (Figure S2.1, Supporting Information). Note that the presence of humidity does not affect the metal work functions,^[^ ^20^ ^]^ as the latter measured by Kelvin probe for Au, Ag, and Al substrates have similar values both in vacuum and in air (40%RH) (Table S2.1, Supporting Information).

**Figure 1:**
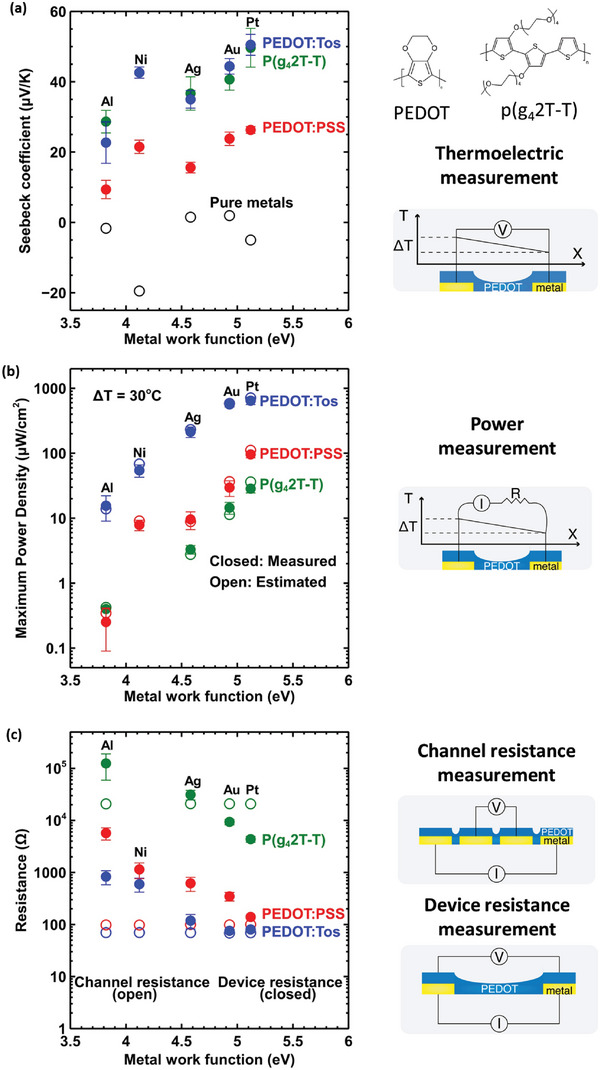
Thermoelectric and electrical characterizations a) The Seebeck coefficient for PEDOT:PSS, PEDOT:Tos and P(g_4_2T‐T) films (closed) plotted against the work function of the metal contact and the Seebeck coefficient of the metals (open).^[^ ^21^, ^22^ ^]^ The PEDOT chemical structure is included as an inset. b) The respective maximum power density for those devices with the comparison between the estimated value (closed) and the measured value (open) at Δ*T* = 30°C. The cross‐section area for our single element devices is 2 × 10^−6^ cm^2^. c) The device (internal) resistance (closed) versus the metal work function for polymer films and the respective channel resistance (open), as measured with a 4‐point probe measurement. Schematics of the measurements are provided on the right side of the plots.

We observe that the Seebeck coefficient of PEDOT:Tos [PEDOT:PSS] increases with the work function of the metal contact, from 22.7 µV K^−1^ [9.4 µV K^−1^] with Al to 50.5 µV K^−1^ [26.3 µV K^−1^] with Pt (**Figure** **1**a) when the measurements are performed in air with 40%RH. This is a large variation achieved just by replacing the metal contact and altering the WF; which suggests that this is not the intrinsic Seebeck coefficient of the PEDOT film, but an “effective” Seebeck coefficient coupled to the energetics at the metal‐PEDOT interface. Note that there is no correlation between the Seebeck coefficients of the metal contacts (open symbols) and the measured Seebeck coefficients (closed symbols), so the important parameter seems to be the position of the Fermi level of the metal. Previous works have reported surprisingly large variations of Seebeck coefficient for both PEDOT:PSS (≈10 µV K^−1^ with Ag contacts^[^ ^1^, ^2^, ^9^, ^12^ ^]^ and ≈20 µV K^−1^ with Au contacts^[^ ^2^, ^6^ ^]^) and PEDOT:Tos (40 µV K^−1^ with Au contacts^[^ ^2^ ^]^). Hence, the large variations in Seebeck coefficient reported in the literature might be due to the different energetics of the chosen metal‐organic interface. Now since there is a correlation between the effective Seebeck coefficient and the metal work function, we need to be aware that for the same type of metal electrode, the effective Seebeck coefficient could also vary slightly, since the effective metal WF – being a surface property – is affected by the metal surface crystallinity,^[^ ^23^ ^]^ morphology, and also by impurities introduced during metal deposition.

Two metal contacts, set at different temperatures (Δ*T* = 1 °C around room temperature) and electrically connected to the conducting polymer film, constitute a single leg OTEG. The generated electrical power can be measured for various load resistances (Figure S1.3, Supporting Information) aiming at finding the maximum output power for *R*_load_ = *R*_dev_. The measured maximum output power density *P*_max_ of the OTEGs based on PEDOT:Tos [PEDOT:PSS] is also increasing with the metal WF, from 0.086 µW cm^−2^ [0.014 µW cm^−2^] with Al to 3.575 µW cm^−2^ [0.525 µW cm^−2^] with Pt (Figure 1b). Note that the measured *P*_max_ (closed symbols) is in rather good agreement with the calculated value based on Equation 1 (open symbols). Furthermore, we investigated that *P*≈Δ*T* ^2^ for our samples (See Section S1, Supporting Information), which results in the power density values for Δ*T* = 30 °C that are measured to be ≈3420 µW cm^−2^ for the Pt/PEDOT:Tos/Pt and the extrapolated value ≈480 µW cm^−2^ for Pt/PEDOT:PSS/Pt (Figure 1b). The maximum power density values cannot be explained solely based on the measured Seebeck coefficients. We find that the device resistance of the polymer legs also depends on the metal WF. For PEDOT:PSS, *R*_dev_ varies from 6 kΩ for Al to 0.15 kΩ for Pt (Figure 1c), while for PEDOT:Tos *R*_dev_ decreases from 0.7 kΩ (Al) to 0.01 kΩ (Pt). Since the channel resistance, measured with the 4‐probe method, is constant for the various films (Figure 1c), we attribute the change in *R*_dev_ to the variation of the contact resistance *R*_c_ with the metal contact (Figure 1c), as the device resistance should be the sum of the channel resistance with the contact resistances. In order to provide further evidence on that, we extracted *R*_c_ values for the PEDOT:PSS/Ag contact (6.7 ohm cm) and PEDOT:PSS/Au contact (2.4 ohm cm) and observed a similar dependence for the total device characteristics.

Interestingly, the interfacial phenomenon and its effect on the thermovoltage was also found for the polar polythiophiophene copolymer, P(g_4_2T‐T) (≈30 nm thick) used in both electrochemical transistor and thermoelectric applications. This polymer is designed to promote ionic transport with its side chains, it is air stable, easily p‐doped.^[^ ^24^, ^25^ ^]^ Indeed, in Figure 1a we observe that P(g_4_2T‐T):Tos follows the same trend as PEDOT:Tos and PEDOT:PSS with the various metals, reaching Seebeck coefficients from 28.6 µV K^−1^, with Al, to as high as 49.6 µV K^−1^ for Pt contact. However, due to its high resistance (up to 4 kΩ for Pt, Figure 1c) the resulted output power density is much lower (0.14 µW cm^−2^, Pt contact, Figure 1b) with respect to the one from PEDOT:Tos. Additionally, the behaviour of P(g_4_2T‐T):Tos is similar to PEDOT:Tos in dry and inert air (Figure S1.5, Supporting Information), where its Seebeck coefficient is 23±3 µV K^−1^, which is relevantly close to the value measured with carbon contacts for this polymer in ambient conditions (25±3 µV K^−1^).

Hence, for three conducting polymers, we observe that the WF of the metal contact in an OTEG impacts the resulting Seebeck coefficient, the device resistance, and thus it generated electrical power. In the rest of this manuscript, we decide to focus on the conducting polymer PEDOT, as it is the most commonly used today. We will thus provide evidence and a hypothesis on this device resistance and the effect of the effective Seebeck coefficient based on the polymer/metal energetics.

### 2.2 External Parameters Affecting the Open Circuit Voltage

These findings raise two questions: what is the origin of the interfacial effects and what is the true intrinsic Seebeck coefficient of the polymers? Before designing experiments to answer these questions, it is important to remind that the humidity affects the thermoelectric and electronic properties of PEDOT:PSS. The reason to this is that the thermodiffusion of ions generates an additional contribution to the Seebeck voltage that is time‐dependent and vanishes after long time.^[^ ^10^, ^26^ ^]^ In our measurements, carried out at 40%RH, the Seebeck coefficient is constant over an extended period of time (Figure S3.1, Supporting Information), thus indicating the absence of an ionic Seebeck effect. Moreover, there is no additional static voltage as indicated by the fact that the intercept of the thermovoltage versus Δ*T* curve runs through zero (see Figure S1.2, Supporting Information). The voltage contribution from the ionic Seebeck effect in PEDOT:PSS is also known to be negligible at relative humidity levels below 40%,^[^ ^27^ ^]^ and has never been observed in PEDOT:Tos even at elevated humidity levels.^[^ ^9^ ^]^ With this in mind, we then decided to focus on PEDOT:Tos exposed to 40%RH in order to investigate the effect of the metal contact WF. Even though PEDOT:Tos does not display any ionic Seebeck effect, due to its low ionic conductivity, computational microscopy^[^ ^28^ ^]^ supports the presence of water molecules within the film at ambient conditions, which could potentially trigger an interfacial redox reaction.

**Figure 2:**
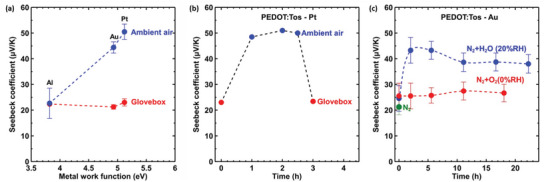
Thermoelectric characterization under different atmospheric conditions:a) The Seebeck coefficient of PEDOT:Tos fabricated and measured inside the glovebox, in comparison with ones that were fabricated and measured in air. b) The Seebeck coefficient of PEDOT:Tos/Pt fabricated and measured inside the glovebox, depicted as it is exposed to air and returned back to the glovebox. c) The Seebeck coefficient of PEDOT:Tos/Au under various atmospheric conditions.

PEDOT:Tos films were deposited inside a glovebox in an inert and dry atmosphere ([O_2_] = 3.9 ppm, [H_2_O] = 3 ppm). The Seebeck coefficients of PEDOT:Tos/Al, PEDOT:Tos/Au, and PEDOT:Tos/Pt were not dependent on the metal WF, and reach 21±1 µV K^−1^ at room temperature, thus matching previously reported values for experiments conducted under the same conditions (**Figure** **2**a).^[^ ^4^ ^]^ We believe that this value is the true intrinsic Seebeck coefficient of the PEDOT:Tos films (i.e., without any contribution introduced by the metal contact) obtained at high oxidation level (30%) typically obtained by the iron oxidant in the polymerization synthesis. In order to provide further evidence of this statement, we measured the Seebeck coefficient of PEDOT:Tos, and PEDOT:PSS films with contacts made of printable carbon paste (WF = 4.7 eV; FigureS2.2, Supporting Information); the respective values were 21±2 and 17±1 µV K^−1^, which are indeed similar to the ones measured in the dry and inert atmosphere. The carbon electrodes have no surface oxide and are electrochemically inactive in the range of electrical potential considered in the measurements (<0.5 mV). When the samples were exposed to ambient atmosphere (40%RH at room temperature), the Seebeck coefficient increased from 21 to 50 µV K^−1^ for OTEGs with Pt contacts (Figure 2b). Interestingly, when the samples were re‐introduced into the glovebox, the Seebeck coefficient returned back to its original value of 21 µV K^−1^. Next, we identified whether the relative humidity and/or the presence of oxygen were key parameters to affect the effective Seebeck coefficient. We conducted the thermoelectric measurements in three different atmospheres: dry nitrogen, dry oxygen‐nitrogen mixture and humid nitrogen. After transferring the samples to a glovebox filled with dry air (O_2_+N_2_, −30 °C dew point), we observed a slight increase in the Seebeck coefficient value reaching 25±3 µV K^−1^ (Figure 2c). One set of samples was then kept in dry nitrogen atmosphere while it was characterized over an extended period. These OTEGs displayed a constant open circuit voltage over the entire measurement period. Another set of samples was instead exposed to humid nitrogen and underwent an immediate and rapid increase of the effective Seebeck coefficient, starting at 25 µV K^−1^ and finally reaching 43 µV K^−1^ after 20 h of exposure. After 20 h, the exposure to humidity was terminated and dry nitrogen was fed into the chamber. We then observed that the Seebeck coefficient slowly reduced toward its original value of 21 µV K^−1^. The power output recorded from this Au/PEDOT:Tos/Au OTEG leg also displays an evolution that strongly depends on the relative humidity level (Figure S1.3, Supporting Information). Consequently, we can then conclude that humidity is a key‐parameter to observe the effect of WF on the measured Seebeck coefficient for PEDOT films.

### 2.3 Probing the Buried Interface

**Figure 3:**
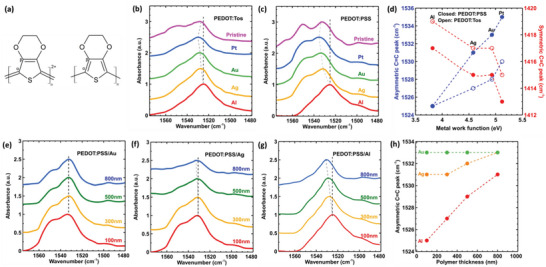
Spectroscopic characterization of the polymer‐metal interface: a) Schematic representation of PEDOT in the quinoid (doped) (left) and benzoid (undoped) (right) forms. b) IRAS absorption spectra of PEDOT:Tos and (c) PEDOT:PSS films (≈100 nm thick) on various metal substrates: Al, Ag, Au, and Pt. ATR‐FTIR spectra if pristine PEDOT was measured on CaF_2_. We show here the evolution of the assymetric C=C peak for those systems (dashed line), while the full spectra are provided in the supplementary information. d) Evolution of the asymmetric and symmetric C=C band maxima of the thiophene ring for PEDOT:PSS (closed) and PEDOT:Tos (open) on the various metals. e–g) IRAS spectra of PEDOT:PSS of varying thicknesses on e) Au, f) Ag, and g) Al. We show here the evolution of the asymetric C=C peak for those systems (dashed line, full spectra in supplementary information). h) Evolution of the asymmetric and symmetric C=C band maxima of the thiophene ring for PEDOT:PSS of various thicknesses on Au, Ag, and Al.

Vibrational spectroscopy is known to be sensitive to the oxidation level of PEDOT^[^ ^29^, ^30^, ^31^ ^]^ and can thus be used to probe the metal‐PEDOT interface. To identify the intrinsic vibrational transitions of the polymer, we first measured ATR‐FTIR (attenuated total reflectance Fourier transform infrared spectroscopy) of PEDOT:PSS and PEDOT:Tos films on a non‐conducting IR‐transparent CaF_2_ substrate (**Figure** **3**b,c and SI, Section 4). Two important vibrational transitions are the asymmetric C=C stretching mode doublet of the thiophene ring located at 1533 & 1547 cm^−1^ and its symmetric C=C counterpart at 1415 cm^−1^.^[^ ^32^ ^]^ Next, infrared reflection absorption spectroscopy (IRAS)^[^ ^33^, ^34^, ^35^ ^]^ was used to probe changes in those vibration bands induced by a modification in the oxidation level at the metal‐PEDOT interface. From the IRAS spectra for PEDOT:PSS and PEDOT:Tos thin films (thickness ≈100 nm) deposited on top of Al, Ag, Au and Pt (Figure S.4:1‐4, Supporting Information), we focus on the evolution of the frequency of the C=C bonds, see Figures 3b–d. The asymmetric [symmetric] C=C vibration peak maxima of the thiophene unit are located at 1525 cm^−1^ [1417 cm^−1^] on Al, 1530 cm^−1^ [1415 cm^−1^] on Ag, 1533 cm^−1^ [1415 cm^−1^] on Au, and 1535 cm^−1^ [1413 cm^−1^] on Pt. Compared to the pristine PEDOT film, measured on the insulating CaF_2_ substrate, the symmetric peak shift toward lower wavenumbers indicates that PEDOT chains undergo oxidation on the Pt substrate and reduction on the Al substrate.^[^ ^30^ ^]^ This observation is fully consistent with the expected spontaneous electron transfer occurring between the metal and the PEDOT films. Thus, if the polymer WF is smaller than the metal WF (e.g., platinum), the polymer becomes oxidized at the interface^[^ ^15^ ^]^ and the low contact resistance leads to an overall low device resistance and high power (see Figure 1.b,c); while if the polymer WF is larger than the metal WF (e.g., aluminum), the polymer gets reduced at the interface compared to the pristine oxidation state of PEDOT (thus increasing the contact resistance and the overall resistance of the device that limits the generated power, see Figure 1.b,c). Since the asymmetric peak position of the Au sample is the same as with the pristine PEDOT:PSS, there is no apparent interfacial redox reaction. However, there is an interfacial redox reaction between PEDOT:Tos and Au, as the asymmetric peak of the pristine PEDOT:Tos is lower than that of PEDOT:Tos/Au, which is likely due to the lower WF of PEDOT:Tos compared to PEDOT:PSS.

In IRAS, the incident infrared light is reflected at grazing incidence by the metal surface so that the absorbed wavelengths correspond to transitions in the vibrational modes of PEDOT at the metal interface, as well as in the bulk of the thin film. Tuning the thickness of the PEDOT film provides us with a probe to study the volumetric extension of this redox phenomenon. When there is no interfacial redox reaction, as identified for the PEDOT:PSS/Au system, the peak positions remain constant while increasing the polymer thickness from 100 to 800 nm, although we do observe a slight trend in the peak ratios of the asymmetric C=C doublet. In contrast, the C=C_asym_ peak of PEDOT:PSS on Al [Ag] varies with the thickness from 1525 cm^−1^ [1531 cm^−1^] for a 100 nm thick film to 1531 cm^−1^ (1533 cm^−1^) for a 800 nm thick film (Figure 3e—h; Figure S4.5, Supporting Information). This supports the formation of an oxidation level profile within the PEDOT film that extends from the metal‐interface into the polymer bulk, reaching up to several hundreds of nanometers of thickness.

**Figure 4:**
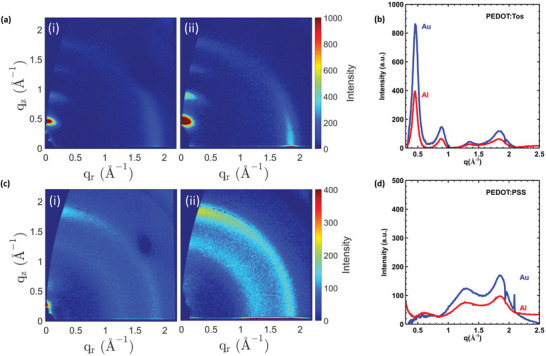
Morphological characterization of the polymer‐metal layers a) The 2D background subtracted scattering images for PEDOT:Tos on Al (i) and Au (ii). b) The 1‐D scattering patterns for PEDOT:Tos on Au and Al. c) The 2D background subtracted scattering images for PEDOT:PSS on Al (i) and Au(ii). d) The respective 1D scattering patterns for PEDOT:PSS on Au and Al.

Electrochemical doping/dedoping of PEDOT thin films is known to alter its crystalline structure.^[^ ^36^ ^]^ We studied changes in morphology, induced by the oxidation level profile triggered by the redox interfacial chemistry, by using grazing incidence wide angle X‐ray scattering (GIWAXS). As the metals themselves have a strong scattering contribution, we deposited a very thin layer (10 nm) of Au and Al on Si (with native oxide) followed by the deposition of the PEDOT films on top. 2D GIWAXS scattering patterns (Figure S6.1) were recorded and a background (Si + metal) subtraction was carried out. This allowed us to decouple the pure PEDOT contribution (**Figure** **4**), which was also integrated to obtain the 1D scattering patterns. The scattering features observed for both PEDOT:Tos and PEDOT:PSS agree well with those previously reported in literature.^[^ ^36^, ^37^ ^]^ Interestingly, a higher degree of order (i.e., a higher integrated (100) peak intensity) is observed for PEDOT in contact with Au as compared to Al, which is consistent with the higher oxidation level of PEDOT:Tos on Au.^[^ ^36^ ^]^

## 3 Discussion

### 3.1 Thermoelectric Properties of the Pristine PEDOT:Tos

First, the thermoelectric properties of a material are summed up in the thermoelectric figure of merit *ZT* ( = *σ S* ^2^ T/*κ*) that is correlated to the theoretical heat‐to‐electricity conversion efficiency of the material. Here we demonstrate that the thermoelectric properties of the single‐leg thermoelectric device “metal/PEDOT/metal” is affected by a charge transfer reaction taking place right at the metal/PEDOT interface. Indeed, the bulk resistance of the film measured by 4‐point probe (Figure 1c, empty symbols) does not vary with the metal contact; while the total resistance of the single‐leg device (Figure 1c, filled symbols) is affected by the metal/PEDOT contact resistance and correlated to the workfunction of the metal. The result of this interfacial effect is not only a change in the overall resistance of the thermoelectric leg metal/PEDOT/metal but also an effect on its thermo‐induced voltage (Δ*V*/Δ*T*, Figure 1a); thus affecting the power factor and the generated electrical power density (Figure 1b).

But to set the ground, we first summarize what is known about these PEDOT:Tos thin films without considering the interfacial effect. The FTIR spectrum in Figure 3b (pink) is recorded for the pristine PEDOT film measured on the insulating CaF_2_ substrate, that is without the interfacial effect with a metal. The frequency of vibration of the thiophene ring in PEDOT is known to be correlated to its oxidation level and conductivity. The position of the vibrational asymmetric C=C vibration peak maximum of the thiophene unit is located at 1533 cm^−1^; which corresponds to PEDOT films of conductivity ≈500 S cm^−1^ and an oxidation level of ≈30%. ^40^ The corresponding typical charge carrier mobility and charge carrier concentration are of the order of 5 × 10^−4^ cm^2^ Vs^−1^ and 1.7  ×  10^21^ carriers cm^−3^.^[^ ^38^, ^39^ ^]^ PEDOT has been demonstrated to follow Wiedemann–Franz law with a Lorentz number larger than the metal gas.^[^ ^4^ ^]^ For a PEDOT:Tos layer of conductivity of 500 S cm^−1^, the phonon contribution to the thermal conductivity is ≈0.8 Wm^−1^K^−1^ and the electronic contribution is ≈0.7 Wm^−1^K^−1^ for a total thermal conductivity of 1.5 W m^−1^ K^−1^ at room temperature.^[^ ^38^ ^]^ The Seebeck coefficient of the PEDOT:Tos thin film measured in inert and dry atmosphere is ≈21 µV K^−1^ that is neglecting the effect of metal contact found in this work upon exposure to humid air. Hence, the ZT value of the PEDOT:Tos film is ≈0.007 at room temperature. It is important to mention that the purpose of this work is not to provide the highest ZT value for a PEDOT:Tos film, for that it is known that the oxidation (doping) level should be decreased,^[^ ^5^ ^]^ but to demonstrate how the metal contact can actually change the thermoelectrically generated electrical power from a single leg of the following architecture: metal/PEDOT:Tos/metal. This highlights that ZT is not actually enough to properly characterize the thermoelectric performance of organic thermoelectric devices, but that interfacial engineering appears to be an additional optimization strategy to improve the generated electrical power density.

### 3.2 Generated Electrical Power Density of Metal/PEDOT/Metal Devices

**Figure 5:**
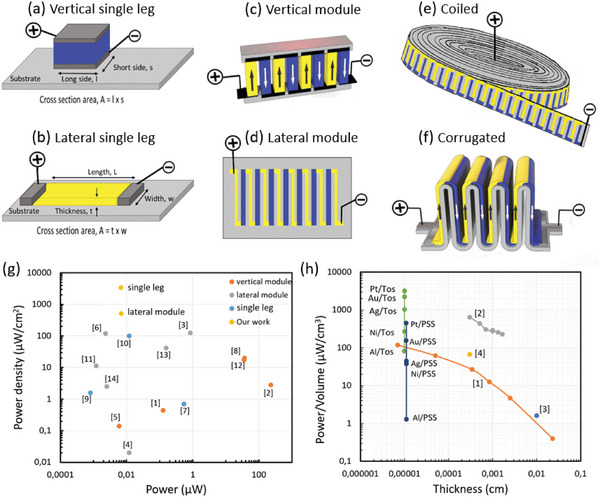
Various architectures for thermoelectric modules and comparison of generated power. a) Vertical single leg; b) lateral single leg; c) vertical module^[^ ^40^ ^]^; d) lateral module; e) lateral module coiled into a vertical module^[^ ^40^ ^]^; d) lateral module folded into a vertical corrugated module^[^ ^41^ ^]^ . g) Comparison of the maximum power density for Δ*Τ* = 30 °C for single leg (blue symbols), vertical modules (orange symbols), lateral modules (gray symbols), and our result for Pt/PEDOT:Tos/Pt single leg and the Pt/PEDOT:PSS lateral module (yellow symbols). The data from the literature are extrapolated and summarized in Table S1.1 (Supporting Information). Given that power density follows a parabolic law of *P*≈ Δ*T* ^2^, we converted the various values into power densities (following the cross‐section area and area occupancy/fill factor) and extrapolated at Δ*Τ* = 30 °C. The results for the high temperature power measurements for our samples are presented in Section S1 (Supporting Information). h) Power per volume versus film thickness for our metal/PEDOT(Tos or PSS)/metal single lateral leg OTEGs. Also the power per volume of other single PEDOT leg in lateral configuration are reported from the literature in the following references: [1] = ref. [43]; [2] = ref. [8]; [3] = ref. [44]; [4] = ref. [45].

A thermoelectric leg is composed of a thermoelectric material sandwiched between two metal contacts. It constitutes the elementary unit of a thermoelectric generator. There are two kinds of leg architectures, either vertical (**Figure** **5**a) or lateral (Figure 5b). Thermoelectric modules can be fabricated by replicating the legs electrically in series and thermally in parallel to amplify the voltage and electrical power. In homopolar modules, e.g. a p‐type module, the p‐type thermoelectric legs are connected with a conductor that possesses a Seebeck coefficient close to zero (Figure S1.11, Supporting Information); while in bipolar modules, both n‐type and p‐type legs are used to form either vertical modules (Figure 5c) or lateral modules (Figure 5d). The most used architecture in the field of thermoelectricity is the vertical module, but the possibility to use printing techniques to pattern many thermoelectric legs at low cost and on large surface area has led to new thinking regarding the use of lateral modules. Here, organic materials, although poor thermoelectrics compared to the best inorganics, appear unique as they can be solution processed at low temperature and are mechanically flexible. Various thin‐film OTEGs in a lateral geometry have been proposed to be combined with low‐cost printing and coating techniques^[^ ^38^, ^39^ ^]^ and brought into a vertical architecture by a judicious choice of device construction, such as i) the coiled‐up TEG architecture (Figure 5e)^[^ ^40^ ^]^; ii) the corrugated architecture (Figure 5f)^[^ ^41^ ^]^ obtained by folding; or iii) the laminated architecture.^[^ ^42^ ^]^

In this work, we have focused on the elementary lateral single leg OTEG, we have demonstrated that for the same material ZT, the lateral OTEGs generate an electrical power that vary over three orders of magnitude just by changing the metal contact in the metal/PEDOT/metal device (Figure 1b). It is desirable to compare our work to the state of the art in the literature. However, since we are not interested in the ZT of the materials but the power of the device, we found no standard way to report the data. The most common way is to report the power density of a OTEG defined by the power generated divided by the cross‐section area A of the device (*P*_max_/A); together with the fill‐factor (i.e., the fraction of the area cross section occupied with the thermoelectric material). The cross‐section area is indicated in Figure 5a,b for the vertical and lateral single legs. In a lateral single leg, a thick thermoelectric film and a thin substrate should lead to high fill‐factor, and thus improved power per surface area: indeed, the inactive substrate is just leaking the heat and does not provide power. Taking the power per cross‐section area of the PEDOT thin‐film leg and neglecting the cross section of the substrate is equivalent to an estimate of the maximum theoretical power value of a one‐leg lateral TEG for a fill factor of 100% (FF = 1). For the OTEG modules based on PEDOT found in the literature, we extrapolated the value of power density (µW cm^−2^) for Δ*T* = 30 °C and FF = 1. This offers a first strategy to compare the generated power from modules and single leg generators (see the last column in the Table S1.1, Supporting Information). We thus plot the extracted power density versus the measured power of the OTEGs for the data found in the literature in Figure 5g. The modules that give highest power are the vertical modules (orange symbols) since their cross‐section area is largest; while the largest power densities are for the lateral modules (gray symbols). This last observation relates to some anisotropy in the PEDOT legs that is discussed in the next paragraph. As a research community, we need to direct our effort to the formation of high power and high‐power density OTEGs (toward the top right corner of Figure 5g). Our best single leg device Pt/PEDOT:Tos/Pt, is stable in air and upon heating cycles (Figure S1.9,10, Supporting Information), and provides the highest power density of 3420 µW cm^−2^ but it possesses low power as the film is extremely thin. We made also a simple demonstration of a homopolar lateral module with 7 PEDOT:PSS legs (t = 200 nm, l = 20 mm, w = 2 mm) and 8 Pt thin film connectors. The module has a fill factor FF = 0.6, generated 2 mV at Δ*T* = 10 °C (Figure S1.11, Supporting Information) and a power density of 0.34 µW cm^−2^ at Δ*T* = 1 °C, which is extrapolated to 514 µW cm^−2^ for Δ*T* = 30 °C and FF = 1 (yellow symbol in Figure 5g).

With the understanding that the power generated by an OTEG is not only dependent on the ZT of the material but also on the metal contacts, it is relevant way to find a standard the way the generated electrical power from an OTEG could be reported. Our community adopted first the power density by dividing with the relevant cross‐section area (*P*_max_/A). But we might need to go one step further because the maximum power density of lateral single leg OTEG will also depend on the length L of the leg. From Equation1, we have *P*_max_/A = Voc^2^/4A*R*_dev_ with *R*_dev_ = *σ*L/A, where *σ* is the conductivity of the device and L is the device length (Figure 5b). Therefore: *P*_max_/A = Voc^2^/4*σ*L. Hence, one way is thus to report the max power per volume V (*P*_max_/V) where V = AL. In Figure 5h, the volumetric power density *P*_max_/V (in µW cm^−3^) is calculated for L = 1 cm (Δ*T* = 30 °C) and plotted versus the film thickness for lateral thin film OTEGs based on PEDOT found in the literature (see Table S1.2, Supporting Information). The cross‐section area considered is that of the PEDOT film (neglecting the substrate). Since in our OTEGs, the PEDOT films are of the same thickness, the reported powers are points on a vertical line, each point for a specific metal contact. The effect of metal contact is striking as the volumetric power density varies by 3 orders of magnitude for both PEDOT:Tos and PEDOT:PSS lateral single leg OTEGs. Other studies report the power for different thickness of the PEDOT films. It is known that the best thermoelectric properties are obtained for very thin‐films of PEDOT (orange curve) while thick PEDOT layers typically lead to much lower power densities^[^ ^8^, ^43^ ^]^ due to the worse morphology and deterioration of the PEDOT chain *π*–*π* stacking. This explains our previous observation in Figure 5g that vertical modules typically display lower power densities than lateral modules.

### 3.3 Interfacial Energetics and the Effective Seebeck Coefficient

Figure 5h summarizes the dramatic effect of the metal contact on the power generated by the OTEG using the same material (same ZT of the material). In Figure 1, the way *S* and *R*_dev_ of three different conducting polymer layers (PEDOT:Tos, P(g_4_2T‐T):Tos and PEDOT:PSS) depend on the WF of the metal contacts pinpoints that the phenomenon responsible for these measured trends relates to the energetics at the metal‐polymer interface. The measured thermovoltage and the redox chemistry found at the interface between the metal contact and the PEDOT film in OTEG legs is clearly enabled by two factors: i) The WF differences between the metal and the polymer, along with ii) A certain ionic mobility promoted by the presence of humidity. All the metals are used in air. Pt and Au are noble and do not form an oxide layer, while Al, Ag, and Ni are known to form spontaneously a very thin and compact passivating oxide layer (20–40 Å);^[^ ^46^, ^47^ ^]^ which actually prevents against further oxidation and partially protect against corrosion. Interestingly, it has been demonstrated that electrical current can cross those thin oxide layers, such that injection of electronic charges from metal to semiconductor or from semiconductor to metal is possible.^[^ ^48^, ^49^, ^50^ ^]^ Note that the presence of very thin oxide layer (10–20 Å) on low work function metals (Ni, Ag, and Al) could also contribute slightly to the contact resistance (max few Ohms), but the main resistance in Figure 1c is expected to come from the oxidation level of PEDOT in the interfacial chemistry region.

Interfacial dipoles are known to form at the conducting polymer‐metal electrode interface and depend on their WFs.^[^ ^15^, ^51^, ^52^, ^53^, ^54^, ^55^ ^]^ The WF of PEDOT:Tos, P(g_4_2T‐T):Tos and PEDOT:PSS is, respectively, 4.5 ± 0.2, 4.7 ± 0.2 (Figure S2.3, Supporting Information) and 5.0 ± 0.1 eV.^[^ ^56^, ^57^ ^]^ The equalization of the chemical potential of the electrons at the metal‐polymer interface leads to a reorganization of the electronic density (integer charge transfer across the interface) that produces an interfacial dipole.^[^ ^15^ ^]^ If the Fermi level of the metal is higher [lower] than that of the PEDOT film, PEDOT is oxidized [reduced]. The extension of the interfacial dipole into polymer semiconductor films, measured under vacuum conditions, is typically localized over a few nm.^[^ ^15^ ^]^

**Figure 6:**
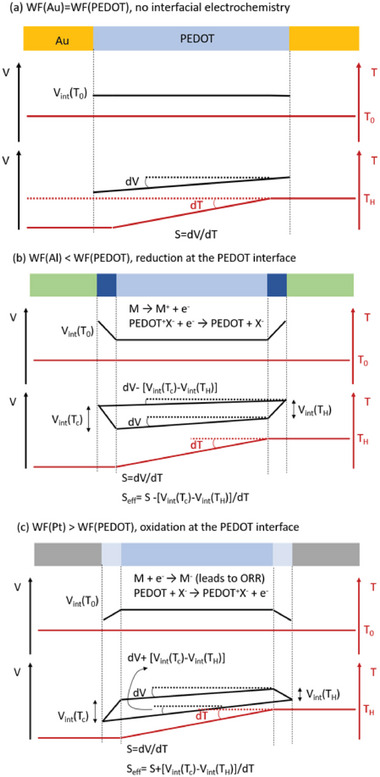
Interfacial effect in the measurement of thermovotlage and Seebeck coefficient. A schematic representation on the effect of polymer/metal energetics on the measured Seebeck coefficient. a) When PEDOT is in contact with a metal with equal work function, b) with a lower work function metal, and c) with a higher work function metal.

When a PEDOT film is exposed to ambient air with 40%RH, ions from the polymer film can potentially diffuse within the polymer since PEDOT is a mixed ion‐electron conductors.^[^ ^58^ ^]^ The presence of mobile ions accompanied by the interface charge transfer (P^+^X^−^ + e^−^ → P^o^ + X^−^), driven by electronic chemical potential equilibration between PEDOT and the metal, is expected to form an inhomogeneous oxidation level profile in the PEDOT bulk starting from the interface. The electronic charge carrier on PEDOT can undergo a diffusion and electronic reorganization within the PEDOT:Tos film through charge carrier hopping: P^+^X^−^ + P^o^ X^−^ → P^o^ X^−^ + P^+^X^−^ (**Figure** **6**e).^[^ ^59^ ^]^ Similar phenomenon can be triggered with an external bias for instance along the channel of an electrochemical transistor.^[^ ^60^ ^]^ These electron transfer processes will eventually result in variations of the PEDOT/metal contact resistance, where an oxidized [reduced] PEDOT volume would have increased [decreased] locally the electrical conductivity.^[^ ^5^ ^]^ Note that even if the first step is the spread of an electronic charge transferred into the polymer, we speculate that a possible next step in the mechanism could be a corrosion in the case of specific metals. Indeed, the charge transfer from a low work function metal to PEDOT:Tos should be accompanied by the accumulation of OH^−^ anions at the vicinity of the metal surface; thus creating a local basic environment. For some metals such as Al and Ag (see Section S13, Supporting Information), the metal oxide dissolves in basic environment, which could then lead to a direct contact between the metal and PEDOT. Such a contact in presence of water, could possibly lead to a dissolution of the metal induced by the charge transfer to PEDOT:Tos according to: Al → Al^3+^ + 3e^−^; P^+^X^−^ + e^−^ → P^o^ + X^−^. Obviously, much more needs to be explored, characterized and understood to strengthen this early hypothesis.

The temperature is expected to affect the concentration of water (evaporation/condensation equilibrium), the charge screening and the degree of advancement of the interfacial redox reaction. Assuming that the interfacial potential drop at the cold side *V*_int_(*T*_c_) is always larger than that at the hot side *V*_int_(*T*_H_), the description of the electric potential profile along the metal‐polymer‐metal OTEG leg, submitted to a temperature gradient, should include the drop in Seebeck potential within the PEDOT (*S* = d*V*/d*T*) film, as well as the two dissimilar interfacial potential drops located at the hot (*V*_int_(*T*_H_)) and cold sides (*V*_int_(*T*_c_)). With such a description (Figure 6), the temperature dependence of the interface potential drop *V*_int_(*T*) = *f*(*T*) dictates if the measured Seebeck coefficient is larger or smaller than the true Seebeck coefficient of the polymer, through *S*_eff_ = *S_mat_* ‐[*V*_int_(*T*_c_)‐*V*_int_(*T*_H_)]/d*T*. For a metal with a work function lower than PEDOT:Tos, there is an interfacial reduction of the polymer that decreases its Fermi level^[^ ^61^, ^62^ ^]^; while the opposite is for a metal with higher work function than PEDOT:Tos. Hence, without temperature gradient, the potential profile along the OTEG legs differs (Figure 6b vs Figure 6c). For a metal with lower work function than PEDOT:Tos, it is expected that the effective Seebeck coefficient *S*_eff_ = *S* ‐[*V*_int_(*T*_c_)‐*V*_int_(*T*_H_)]/d*T*; that is the effective Seebeck coefficient measured is smaller than the true Seebeck coefficient of the material. On the contrary, for a metal with higher work function than PEDOT:Tos, *S*_eff_  > *S*. This possible explanation is in agreement with the observation for the measured values of the Seebeck coefficient of PEDOT:Tos with various metal electrodes (see Figure 1a).

Dissimilar metallic contacts have suggested a modification of the Seebeck coefficient by surface polarization effects.^[^ ^63^ ^]^ In our case, OTEGs based on different metal contacts, as in the case with Pt/PEDOT:PSS/Ag (*S*_meas_ = 29.5 µV K^−1^) and Pt/PEDOT:Tos/Ag (*S*_meas_ = 52.5 µV K^−1^) devices, exhibit higher effective Seebeck coefficient (see Figure S10.1, Supporting Information) than with same metals (see Figure 1). Using different types of contacts could be a new strategy to design specific  *I* versus Δ*T* current‐thermal characteristics for thermoelectric devices like it is done for *I* versus *V* , current‐voltage characteristics in electronics.

## 4 Conclusions

For three different p‐doped polythiophene derivatives (P(g42T‐T):Tos; PEDOT:Tos; and PEDOT:PSS), we observed an enhancement of the effective Seebeck coefficient with the work function of the metal contact. While the resistance of the bulk of the polymer film is unchanged, the contact resistance varies significantly. The conducting polymer at the metal/polymer interface undergoes a local oxidation or reduction depending on the position of the metal Fermi level compared to the electrochemical potential of the *π*‐electronic systems of the polymer. The thermovoltage, arising from this contact phenomenon alone, reaches a magnitude like that of the Seebeck voltage of the conducting polymer itself, thereby providing a major contribution to the resulting thermoelectric performance. That interfacial energetics can severely enhance the output power of OTEGs, which leads to enhanced thermoelectric performance to record‐high OTEG output power densities.

This study opens a new pathway toward the optimization of organic thermoelectric technology. Moreover, our discovery of a doping gradient within the polymer bulk, extending away from the various metal contacts, is of direct relevance for the field of organic electronics in general, as metal‐conducting polymer interface is a fundamental element in many devices. A better understanding of these polymer‐metal interfaces in ambient condition is expected to promote printing technologies in air which is relevant for lateral OTEG that used new architectures such as coiled or corrugated structure to create vertical OTEGs based on thin films, a unique opportunity for organic thermoelectric materials.

## 5 Experimental Section

5.1

5.1.1

5.1.1.1

Details on all processes for material and device fabrication are provided in the Supplementary Information (Section S12, Supporting Information). For all metals the deposition rates were 1 Å s^−1^. Pt was electrodeposited on Au electrodes following the work of Strakosas et al.^[^ ^64^ ^]^ The P(g_4_2T‐T) solutions were deposited on the films through spin coating, and then p‐toluenesulfonic acid was further spin coated to dope the systems.^[^ ^24^ ^]^ The PEDOT:PSS dispersions with 5 vol.% DMSO^[^ ^6^ ^]^ were deposited on the films through spin coating. PEDOT:Tos was polymerized with in situ wet chemical oxidative polymerization with a procedure that is reported elsewhere.^[^ ^56^ ^]^ The 4‐point probe sheet resistance, *R*_s_, was measured with a Keithley 4200. The thickness, *t*, was measured with a Dektak profilometer and the electrical conductivity *σ* was extracted as 1/(*R*_s_ × *t*).^[^ ^65^ ^]^ The thermoelectric measurements were conducted with an in‐lab setup (Figure S1.2, Supporting Information) and details on the device geometry are provided in Supporting Information. The contact resistances were extracted with the transmission line method following a previous report.^[^ ^66^ ^]^ The device geometry is presented in Figure S11.1. (Supporting Information). Details on the IRAS, UPS, GIWAXS, EIS, ICP‐OES, SEM, and Kelvin Probe characterizations are provided in the Supporting Information.

## Conflict of Interest

The authors declare no conflict of interest.

## Supporting information

Supporting Information

## Data Availability

The data that support the findings of this study are available from the corresponding author upon reasonable request.
