# Supplementary material for: Improved Performance of Organic Thermoelectric Generators Through Interfacial Energetics
Source: Adv Sci (Weinh). 2023 May 3;10(20):2206954. doi: 10.1002/advs.202206954 (PMC10369274; doi:10.1002/advs.202206954)
Supplement: Supplementary file 1 — Supporting Information [file ADVS-10-2206954-s001.pdf]

ADVANCED SCIENCE
Open Access

Supporting Information

for *Adv. Sci.*, DOI 10.1002/advs.202206954

Improved Performance of Organic Thermoelectric Generators Through Interfacial Energetics

I. Petsagkourakis, S. Riera-Galindo, T.-P. Ruoko, X. Strakosas, E. Pavlopoulou, X. Liu, S. Braun, R. Kroon, N. Kim, S. Lienemann, V. Gueskine, G. Hadziioannou, M. Berggren, M. Fahlman, S. Fabiano, K. Tybrandt and X. Crispin**

Improved Performance of Organic Thermoelectric Generators through Interfacial Doping Gradients

Petsagkourakis I.[1*†], Riera-Galindo S.[1§], Ruoko T.-P.[1‡], Strakosas X.[1], Pavlopoulou E.[4], Liu X.[1], Braun S.[1] , Kroon R.[1], Kim N.[1], Lienemann S.[1], Gueskine V.[1], Hadziioannou G.[2], Berggren M.[1,3], Fahlman M.[1], Fabiano S.[1], Tybrandt K.[1], Crispin X.[1*]

[1]Laboratory of Organic Electronics, Department of Science and Technology (ITN), Linköping University, SE-601 74 Norrköping, Sweden.

[2]Bordeaux INP, CNRS, Univ. Bordeaux, LCPO, UMR 5629, F-33600 Pessac, France

[3]Wallenberg Wood Science Center, Linköping University, Norrköping, Sweden

[4]Institute of Electronic Structure and Laser, Foundation for Research and Technology, Heraklion, Crete 71110, Greece

[†]Current affiliation : RISE Research Institutes of Sweden, Digital Systems, Smart Hardware, Bio- and Organic Electronics, Bredgatan 33, SE-602 21, Norrköping, Sweden

[§]Current affiliation : Institut de Ciencia de Materials de Barcelona (ICMAB-CSIC), Campus de la UAB, Carrer dels Til lers, s/n, 08193, Bellaterra, Barcelona, Spain

[‡]Current affiliation : Smart Photonic Materials, Faculty of Engineering and Natural Sciences, Tampere University, Tampere 33720, Finland

*corresponding authors

S1: Thermopower measurement schematic, measured raw data, state-of-the-art on organic thermoelectric generator power densities, thermal stability and 7-leg generator.

The shadow masks for this measurement were designed following the guidelines provided by van Reenen & Kemerink on the effect of geometry on the thermoelectric performance, so that the measurements are as accurate as possible from a geometrical perspective[1]. All devices had a cross section area of 100 nm by 1 cm. The temperature difference didn't exceed 3 °C and was measured by two T-type thermocouples. We cross-checked the validity of our setup by measuring Ni and Au foils, following the approach used in Petsagkourakis *et al.*[2].

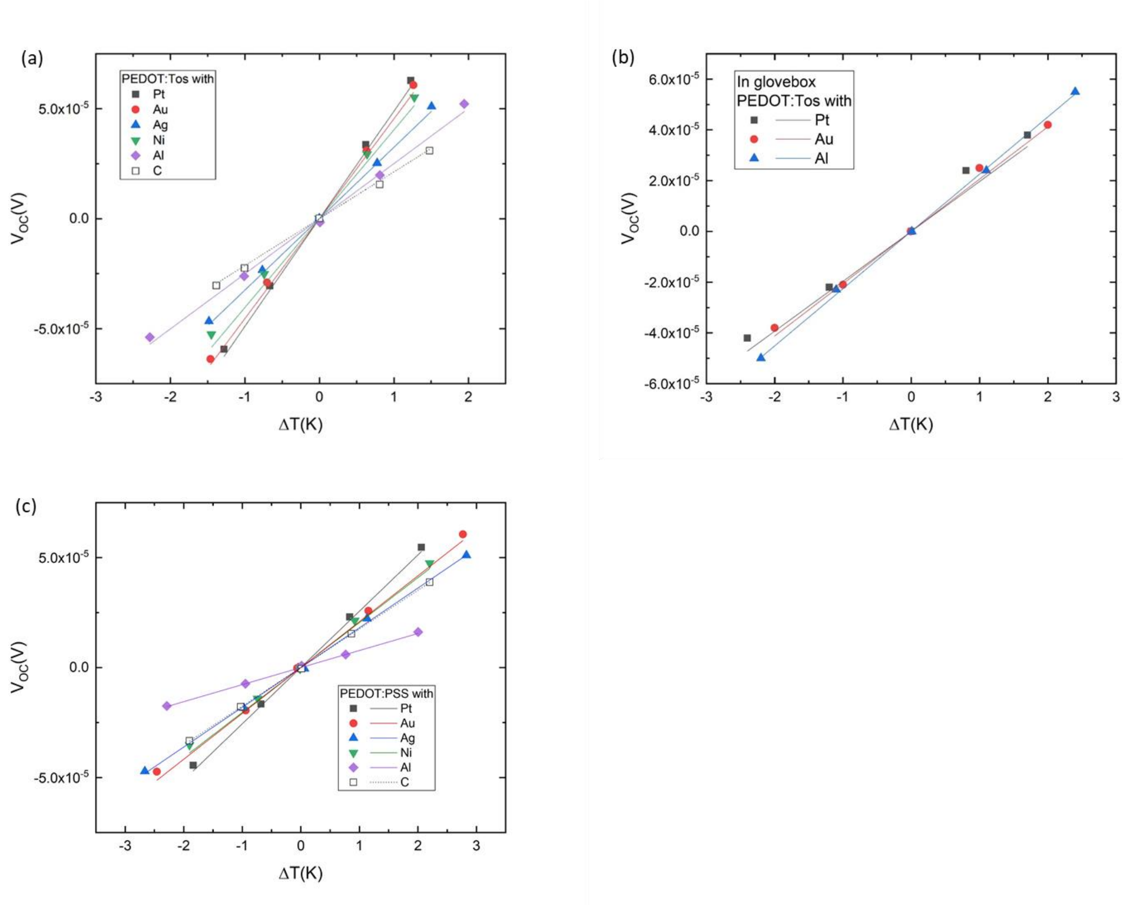

Figure S1.1 : The open circuit voltage V_{OC} plotted against the temperature difference ΔT for (a) PEDOT:Tos under ambient conditions and (b) in dry and inert atmosphere, (c) PEDOT:PSS under ambient conditions, with various metals as the electrodes. In all cases the R^2>0.99.

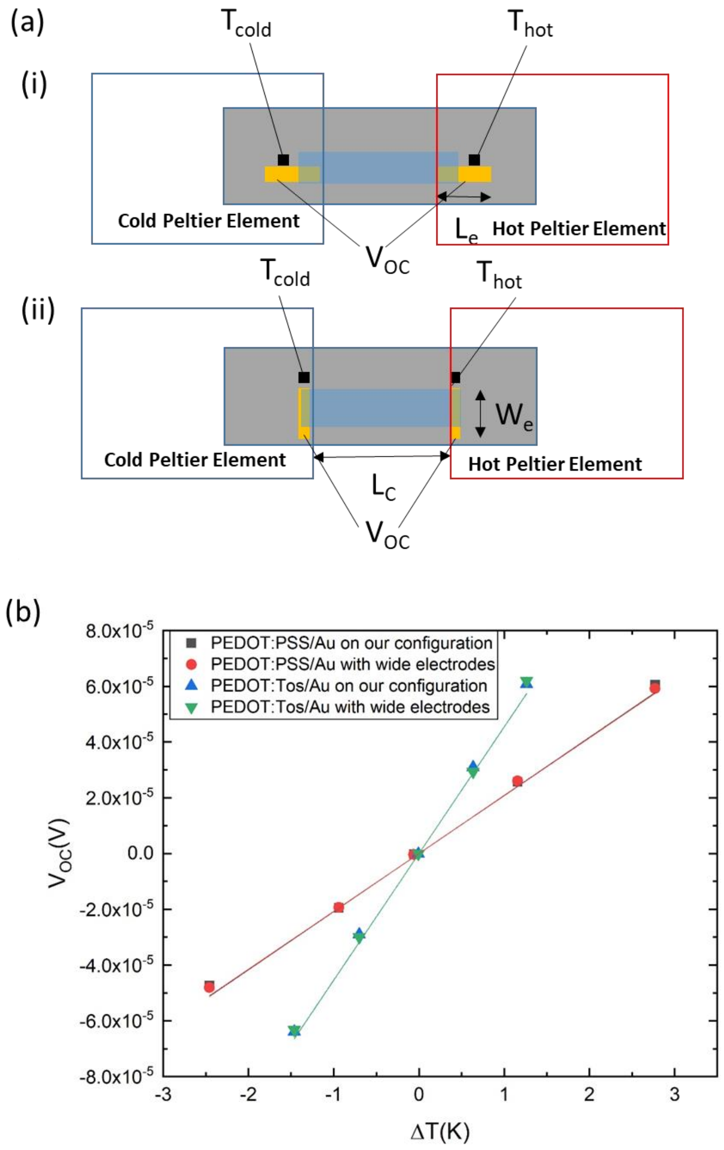

Figure S1.2: (a) Schematic representation of the Seebeck coefficient and thermopower measurement. We put the sample on top of the hot (red) and cold (blue) peltier elements. The sample is on a glass slide (grey) that has the deposited metal (yellow) and polymer layers (transparent blue). The dimensions and positions of the gold electrodes follow the geometrical aspects of Reenen & Kemerink[1] for a trustworthy thermoelectric measurement, where the aspect ratios of the electrodes and the channel should be less than 1. The open circuit voltage was measured with a Keithley nanovoltmeter and the temperature was measured with thermocouples (black) that are attached on the sample with thermally conductive paste (black). We identified that the thermocouple readings were unaffected if the thermocouples were put in a slightly different place (as long as it followed the instructions provide by van Reenen & Kemerink[1]. In order to cross-check the validity of the setup, Au and Ni foils were measured, such as in Petsagkourakis *et al.* [2] Moreover, our reported values for PEDOT:PSS/Au, PEDOT:PSS/Ag and PEDOT:Tos/Au agree with other reported values in the literature[2-5]. To further address the trustworthiness of our devices, we compared two different device geometries (L_C is the distance of the electrodes, L_e and W_e are the length and width of the electrodes): (i) L_C = 10 mm, L_e = 0.1 mm , W_e = 2 mm , while for configuration (ii) L_C = 20

mm, L_e = 0.2 mm , W_e = 4 mm . (b) Comparison of the measured Seebeck for the different setup architectures (our configuration, (i), and wide electrode, (ii)).

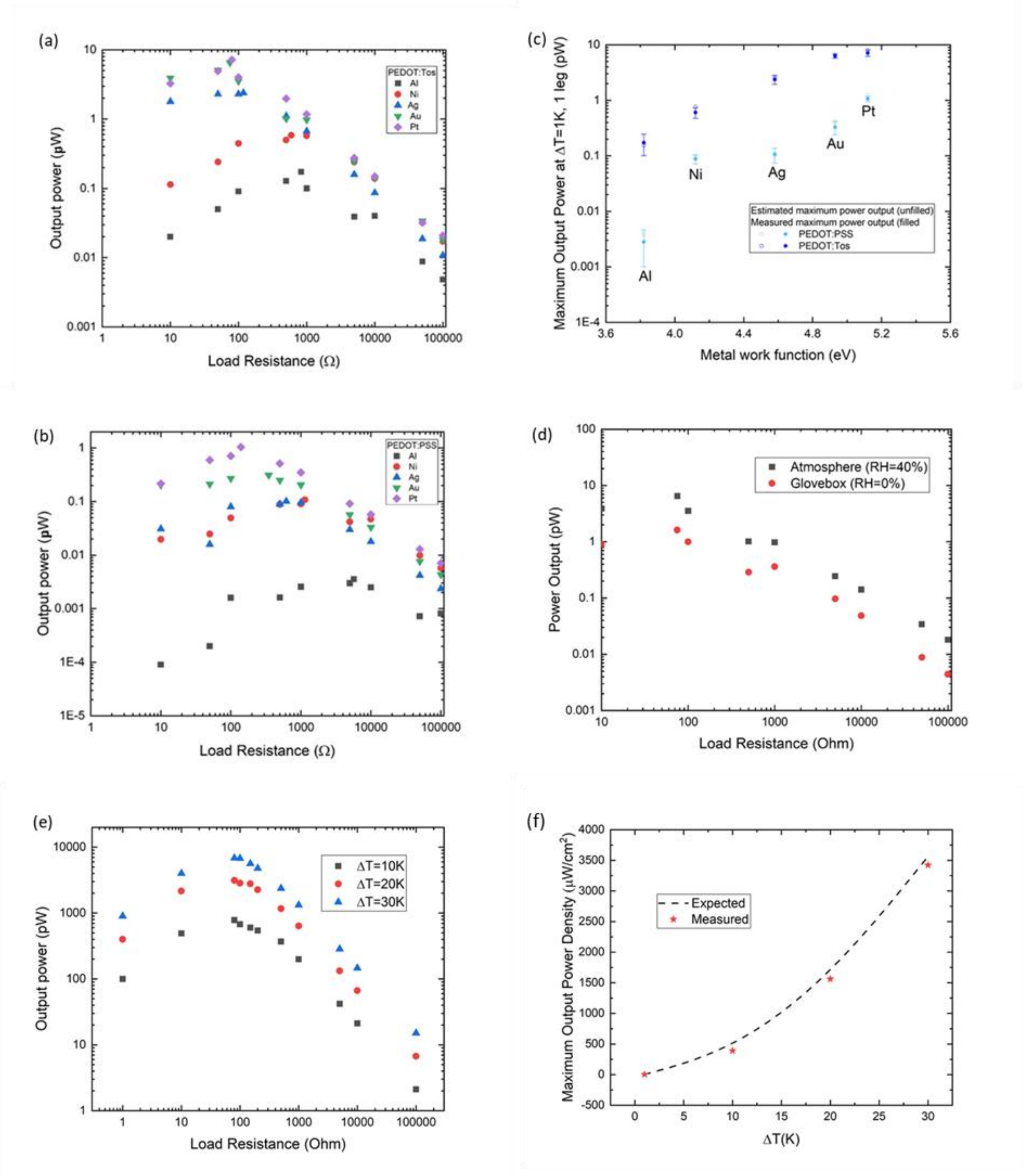

Figure S1.3 : The output power of the PEDOT:Tos (a) and PEDOT:PSS (b) vs. the load resistance. (c) The maximum output power vs. the metal work function. (d) The measured output power vs load resistance for PEDOT:Tos/Pt inside the glovebox (vacuum) and outside the glovebox (ambient air). (e) The measured output power vs load resistance for PEDOT:Tos/Pt for various temperature differences. (f) The dependence of the maximum

output power density to the temperature difference. The samples exhibit the expected P~ΔT^2 tendency.

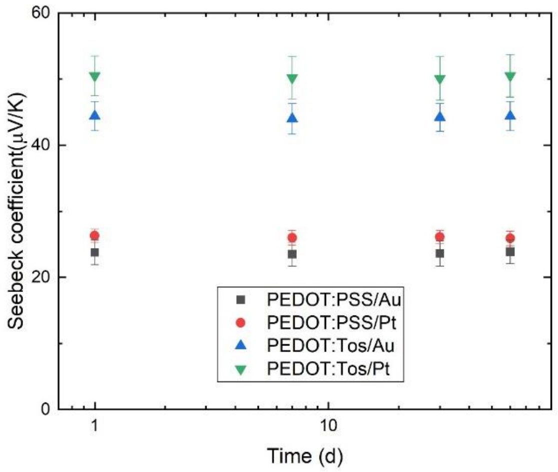

Figure S1.4 : The Seebeck coefficient versus time for the PEDOT:Tos/Au, PEDOT:Tos/Pt , PEDOT:PSS/Au and PEDOT:PSS/Pt samples.

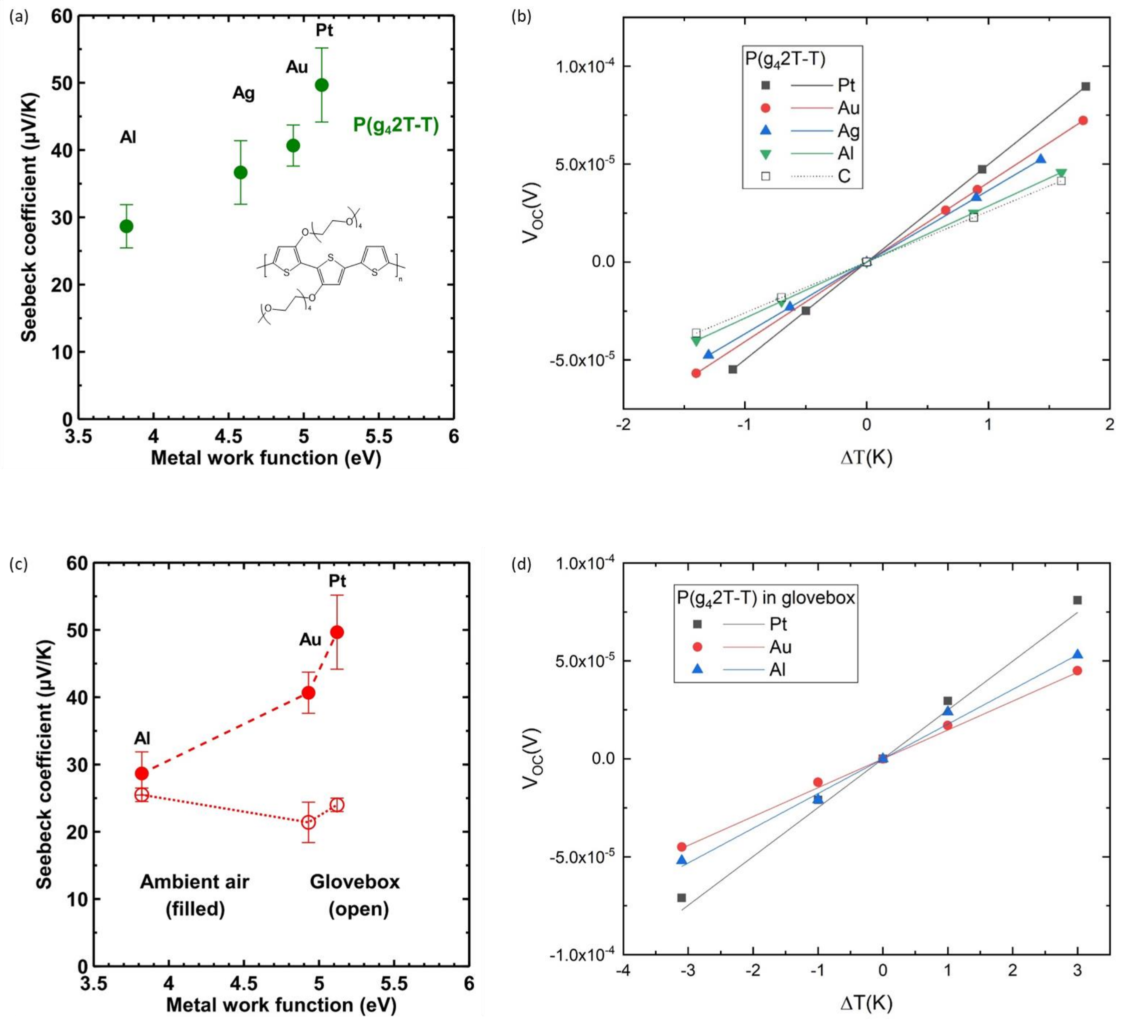

Figure S1.5 : (a) The Seebeck coefficient of P($g_4$2T-T):Tos under ambient conditions with the chemical structure of the copolymer as inset and (b) the respective open circuit voltage vs the temperature difference for various contacts; (c) The Seebeck coefficient of P($g_4$2T-T):Tos in dry and inert atmosphere and (d) the respective open circuit voltage vs the temperature difference for various contacts. In all cases R^2 >0.99.

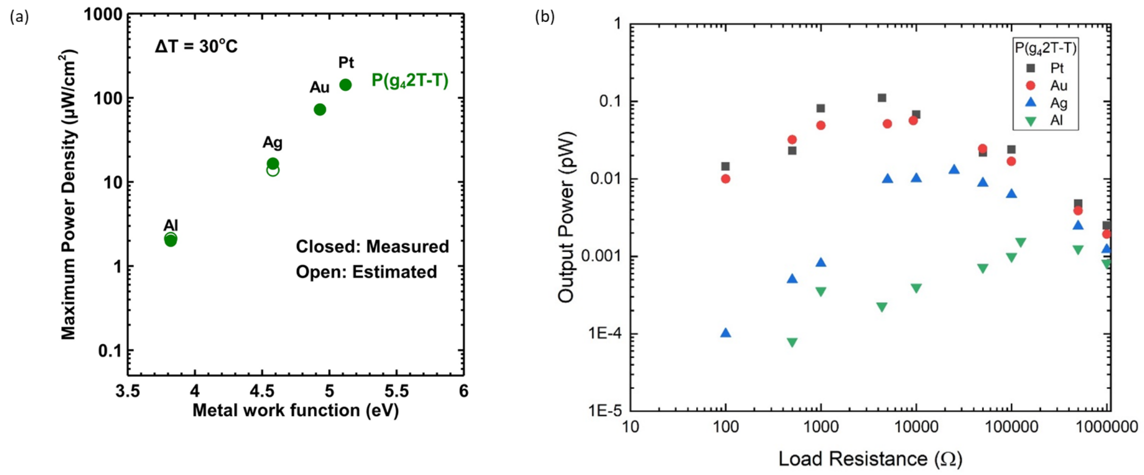

Figure S1.6 : (a) The power density at temperature difference of 30 ^{o}C and the respective (b) output power of the P(g$_4$2T-T):Tos systems vs the load resistance, for the various metals

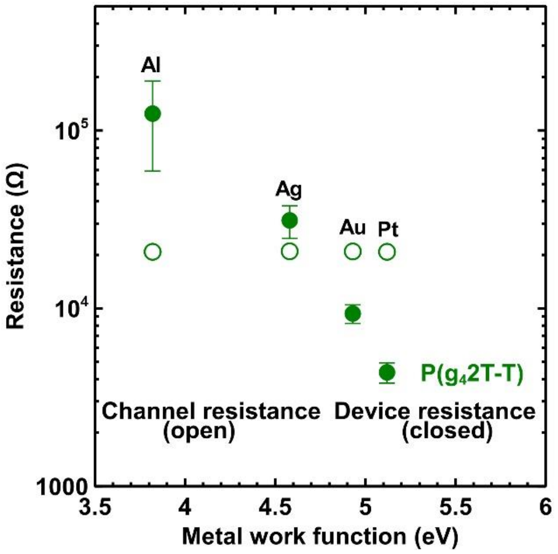

Figure S1.7 : The device and channel resistance of the P(g$_4$2T-T):Tos systems for the various metal contacts.

# in Figure 1	Reference	Power (μW)	ΔT (K)	Cross-section area (A in cm^2) or field factor FF	Architecture	Power density at ΔT ($\mu W/cm^2$)	Calculated power density at ΔT=30 °C ($\mu W/cm^2$) at FF=1
1	[6]	0.128	10	FF=0.47	vertical module	0.023 at FF=0.47	0.44
2	[7]	230	25	FF=0.40	vertical module	0.83 at FF=0.40	2.8
3	[8]	0.85	100	A=$6x10^{-4}$	lateral module	1400 at FF=1	125
4	[9]	0.012	75	A=0.2, FF=0.50	lateral module	0.06 at FF=0.50	0.02
5	[10]	0.006	30	A=0.6, FF=0.07	vertical module	0.01 at FF=0.07	0.14
6	[11]	0.0023	50	A=$7x10^{-6}$	lateral single leg	328	118
7	[12]	0.54	30	A=0.78	vertical single leg	0.7	0.7
8	[13]	37	50	A=1.54 FF=0.84	vertical module	24 at FF=0.84	20.2
9	[14]	0.0008	30	$5x10^{-4}$	lateral single leg	1.6	1.6
10	[5]	0.012	30	A=$1.2x10^{-4}$	lateral single leg	100	100
11	[15]	0.0012	32	A= $1.7x10^{-4}$ FF=0.60	lateral module	6.8 at FF=0.60	11.3
12	[16]	36	50	A=0.9 FF=0.82	vertical module	40 at FF=0.82	17.6
13	[17]	0.157	52	A=$2x10^{-2}$ FF=0.60	lateral module	75 at FF=0.60	41.6
14	[18]	0.0025	30	A= 10^{-2} FF=0.70	lateral module	2.5 at FF=0.70	2.5
PEDOT:Tos/Pt	Our work	0.0068	30	A=$2x10^{-6}$	lateral single leg	3422.5	3422.5

Table S1.1: The power density conversion for the various literature works used in **Fig. 1**. Among the thermoelectric single leg generators, there are two kinds: either lateral or vertical architecture (see **Fig. S1.8**). For each of those types, we extract the power density by using the power generated and calculating the correct cross-section area (see formula below). We report the cross-section area in the **Table S1.1**. Our single leg elements have a thickness of 100nm and width of 2mm , thus a cross-section area of $2x10^{-6}cm^2$. Then we have also projected the

power density for a temperature gradient of 30°C in the last column of the table to be able to compare them all together.

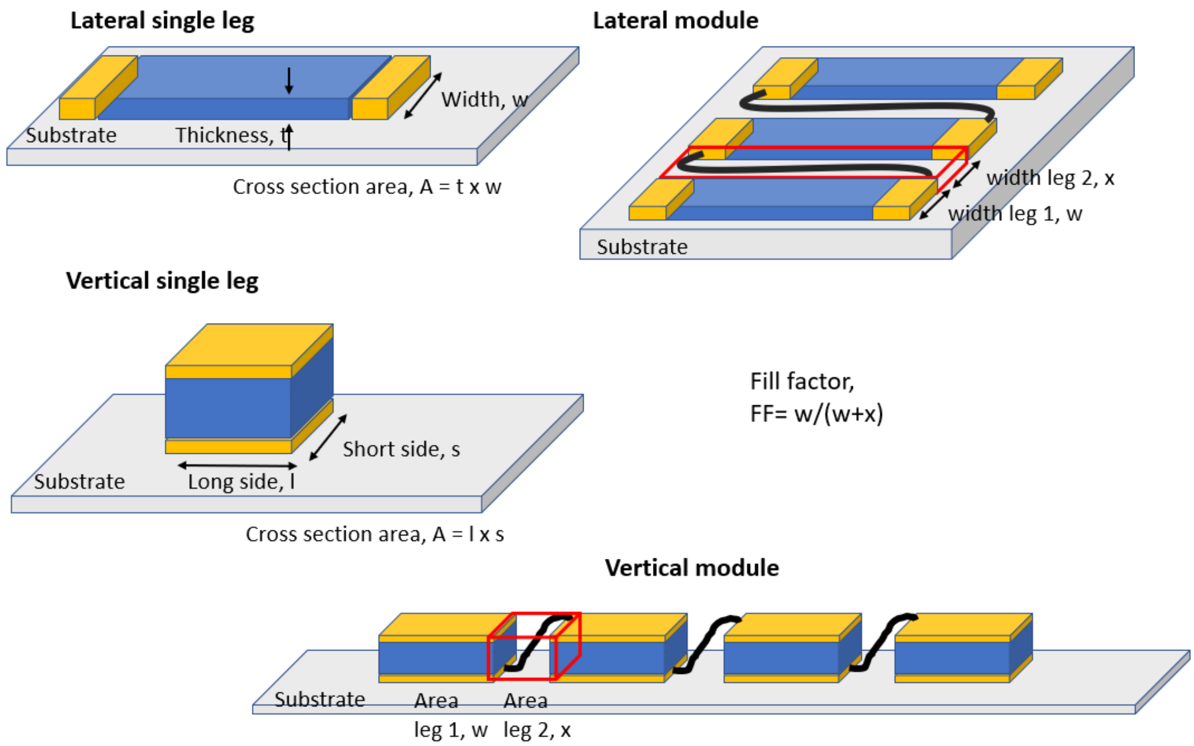

Figure S1.8 : The various architectures for a thermoelectric generator reported in the literature. A lateral single leg, a lateral module, a vertical single leg and a vertical module.The cross-section area is defined as width times thickness for the lateral architectures and as long side times short side for the vertical architectures. Meanwhile, the fill factor of a multi-leg module is defined as the thermoelectric active cross-section area of the whole device.

Among the thermoelectric modules, there are two kinds: either lateral or vertical architecture. Again, we can estimate the cross-section area as well as the fill factor. The fill factor is the part of the area cross-section of the device that is active. For the modules, we extrapolate the value for a fill factor of 100%, which would mean the maximum use of the area. In that way, the power density (μW/cm^2) of a module extrapolated to a fill factor of 100% is a fair comparison to the power density of single leg generators which have by default a fill factor of 100% (FF=1). Hence, the last column of the **Table S1.1** reports the calculated power density for ΔT=30°C, and for at FF=1 for both single leg and modules. We compare those various results in the **Fig. 1d**.

Ref	P (µW)	ΔT (K)	W (cm)	t (cm)	A (cm^2)	L (cm)	P/A@ ΔT (µW/cm^2)	P/A@ L= 1cm; ΔT= 30K (µW/cm^3)
[11]	0.0023	50	1	7 10^{-6}	7x10^{-6}	1	328	118.1
[11]	0.0086	50	1	5 10^{-5}	5 10^{-5}	1	172	61.9
[11]	0.0252	50	1	3.4 10^{-4}	3.4 10^{-}[illegible]	1	74	26.6
[11]	0.0294	50	1	8.4 10^{-4}	8.4 10^{-}[illegible]	1	35	12.6
[11]	0.0332	50	1	2.5 10^{-3}	2.5 10^{-}[illegible]	1	13	4.7
[11]	0.0282	50	1	2.3 10^{-2}	2.3 10^{-}[illegible]	1	1	0.4
[8]	0.85	100	0.4	3 10^{-4}	1.2 10^{-}[illegible]	0.5	7083	637.5
[8]	0.96	100	0.4	5.1 10^{-4}	2.0 10^{-}[illegible]	0.5	4800	432.0
[8]	0.95	100	0.4	7 10^{-4}	2.8 10^{-}[illegible]	0.5	3392	305.3
[8]	1.23	100	0.4	1 10^{-3}	4.0 10^{-}[illegible]	0.5	3075	276.8
[8]	1.29	100	0.4	1 10^{-3}	4.0 10^{-}[illegible]	0.5	3225	290.3
[8]	1.50	100	0.4	1.33 10^{-}[illegible]	5.3 10^{-}[illegible]	0.5	2830	254.7
[8]	1.69	100	0.4	1.66 10^{-}[illegible]	6.6 10^{-}[illegible]	0.5	2560	230.4
[14]	0.0008	30	0.5	0.01	5 10^{-3}	0.1	0.16	1.6
[5]	0.012	30	0.4	3 10^{-4}	1.2 10^{-}[illegible]	1.5	100	66.6
Pt/Tos	0.0068	30±0.05	0.2	10^{-}[illegible]	2 10^{-6}	1	3422.5±83	3422.5 ±
Pt/Tos	7 10^{-6}	1±0.006	0.2	10^{-}[illegible]	2 10^{-6}	1	3.5±0.5	3422.5 ±
Au/Tos	5 10^{-6}	1±0.006	0.2	10^{-}[illegible]	2 10^{-6}	1	3.1±3	2250 ± 47
Ag/Tos	2.3 10^{-6}	1±0.006	0.2	10^{-}[illegible]	2 10^{-6}	1	1.15±0.2	1035 ± 40
Ni/Tos	0.6 10^{-6}	1±0.006	0.2	10^{-}[illegible]	2 10^{-6}	1	0.3±0.07	270 ± 11
Al/Tos	0.18 10^{-6}	1±0.006	0.2	10^{-}[illegible]	2 10^{-6}	1	0.09±0.01	81 ± 6
Pt/PSS	1 10^{-6}	1±0.006	0.2	10^{-}[illegible]	2 10^{-6}	1	0.5±0.06	450 ± 10
Au/PSS	0.34 10^{-6}	1±0.006	0.2	10^{-}[illegible]	2 10^{-6}	1	0.17±0.04	153 ± 8
Ag/PSS	0.1 10^{-6}	1±0.006	0.2	10^{-}[illegible]	2 10^{-6}	1	0.05±0.01	45 ± 3
Ni/PSS	0.085 10^{-}[illegible]	1±0.006	0.2	10^{-}[illegible]	2 10^{-6}	1	0.042±0.008	37.8 ± 1
Al/PSS	0.003 10^{-}[illegible]	1±0.006	0.2	10^{-}[illegible]	2 10^{-6}	1	0.0014±0.0001	1.3 ± 0.2

Table S1.2: The power density conversion for the various single leg lateral devices based on PEDOT-derivatives used in Image 4. Note that the points from ref [8] are power measured on a 5 legs lateral TEG. We did not carry any division by number of legs since it is likely not scaling with number of legs as the internal resistance increase. Hence those data are likely slightly overestimated.

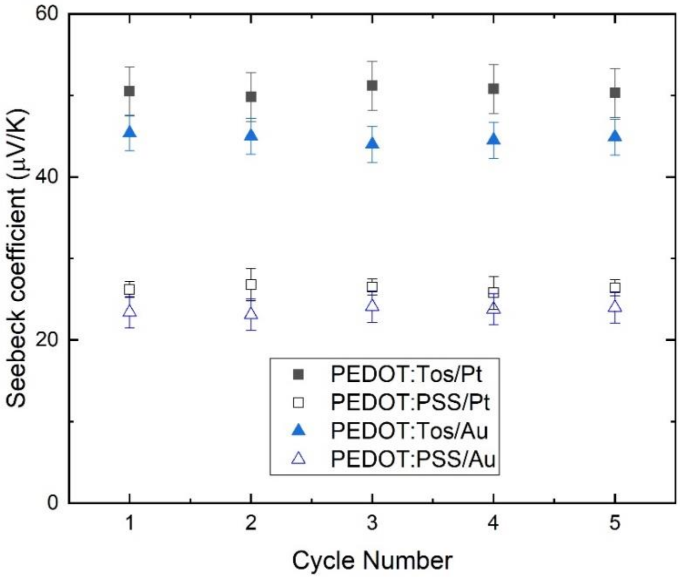

Figure S1.9: Thermal stability of Seebeck coefficient for PEDOT:Tos and PEDOT:PSS samples with Pt and Au contacts , for 5 thermal cycles at 100 ^{o}C, 15mins. The samples were cooled down to room temperature and then measured again.

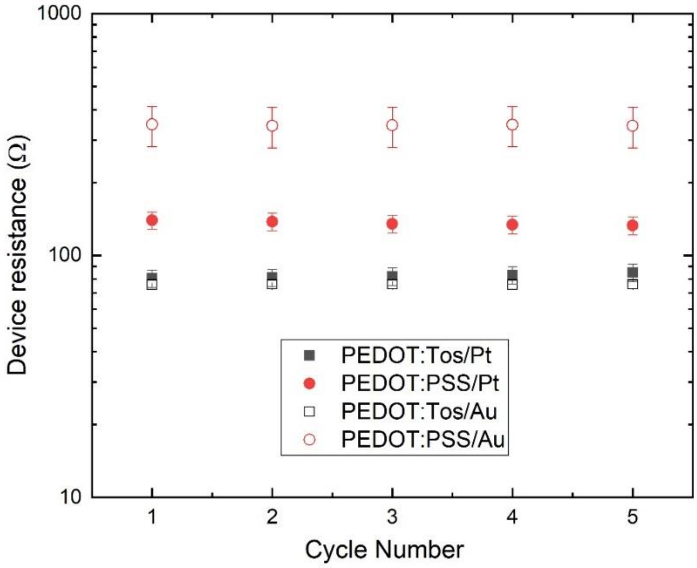

Figure S1.10: Thermal stability of device resistance for PEDOT:Tos and PEDOT:PSS samples with Pt and Au contacts , for 5 thermal cycles at 100 ^{o}C, 15mins. The samples were cooled down to room temperature and then measured again.

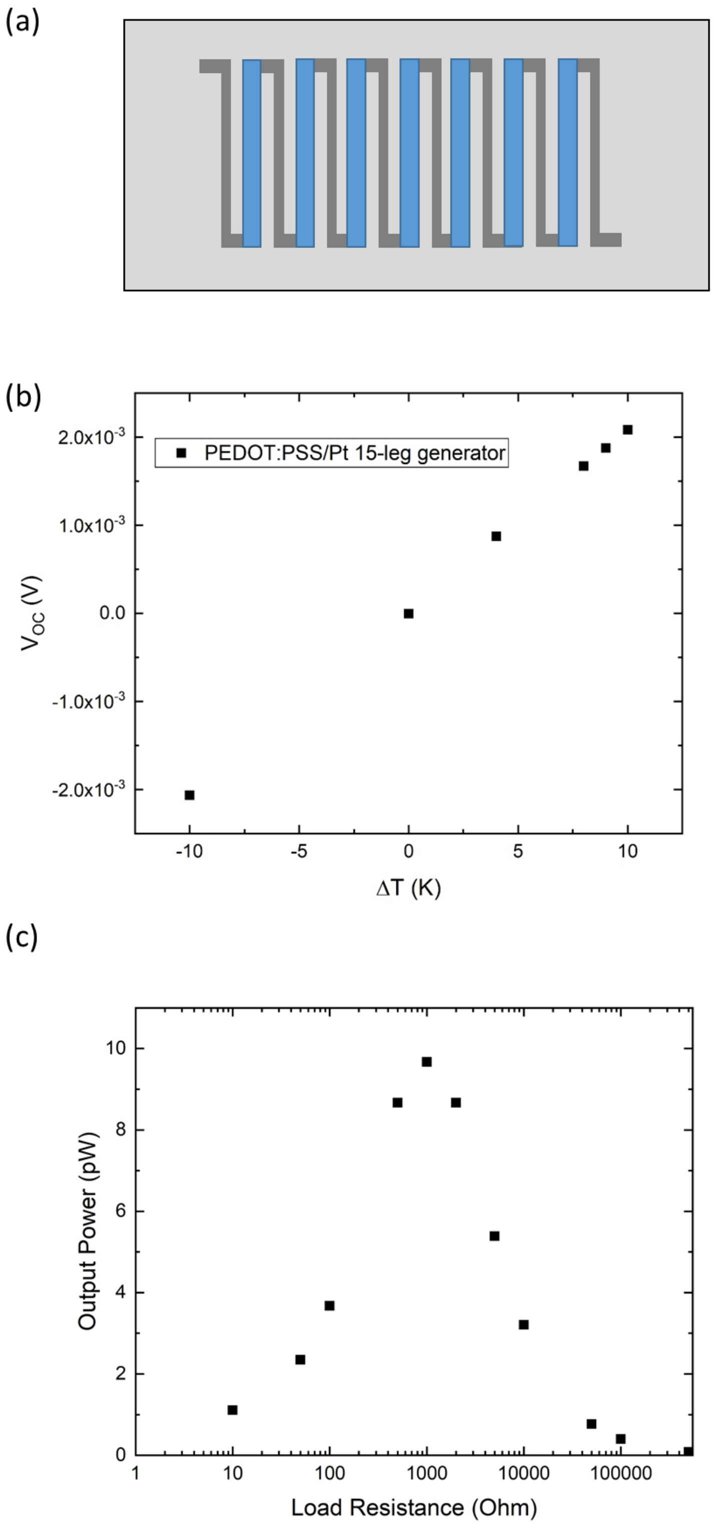

Figure S1.11: (a) Schematic of the 15-leg generator. PEDOT:PSS leg 20mm x 2mm, Pt leg 20mm x 1mm. (b) The output thermovoltage of a 15-leg thermoelectric generator based on the PEDOT:PSS/Pt system comprised of 7 PEDOT:PSS legs and 8 Pt legs (fill factor of 0.6), in temperature differences up to 10 ^{o}C at room temperature. (c) The Output Power vs the Load Resistance of the 15-leg generator at temperature difference of 1 ^{o}C. It should be highlighted that the device resistance is 1000 Ohm which is quite close to the theoretical prediction of 978.6 Ohm for this PEDOT:PSS/Pt multielement module.

S2: UPS spectra and Kelvin probe measurements

The UPS spectra were acquired with an UHV surface analysis system, consisting of an entry chamber (base pressure ~1×10^{-7} mbar), a preparation chamber (~1×10^{-8} mbar), and an analysis chamber (~2×10^{-10} mbar). Monochromatized HeI (hν = 21.22 eV) spectra were recorded with a in-lab designed and built photoelectron spectrometer, and were calibrated by using the Fermi edge and Au $4f_{7/2}$ peak position of an Ar^+ ion sputter-cleaned gold foil. The UPS was performed with an error of ±0.05 eV.

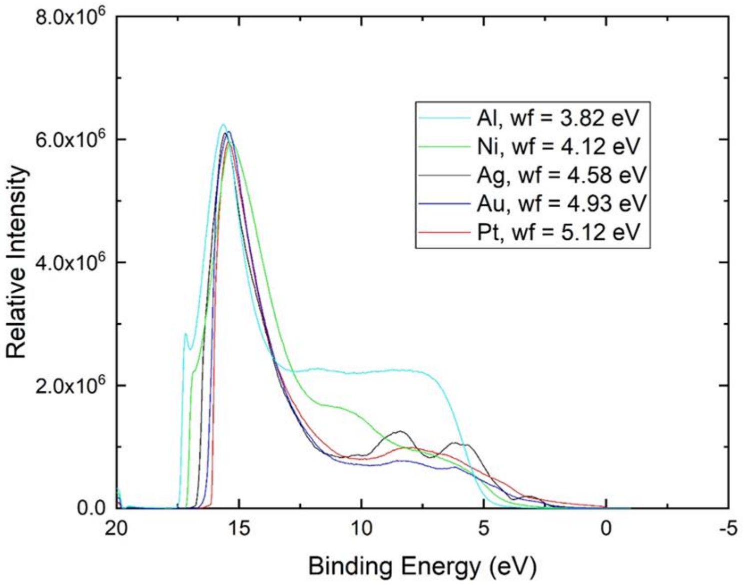

Figure S2.1: UPS spectra for the bare metal substrates

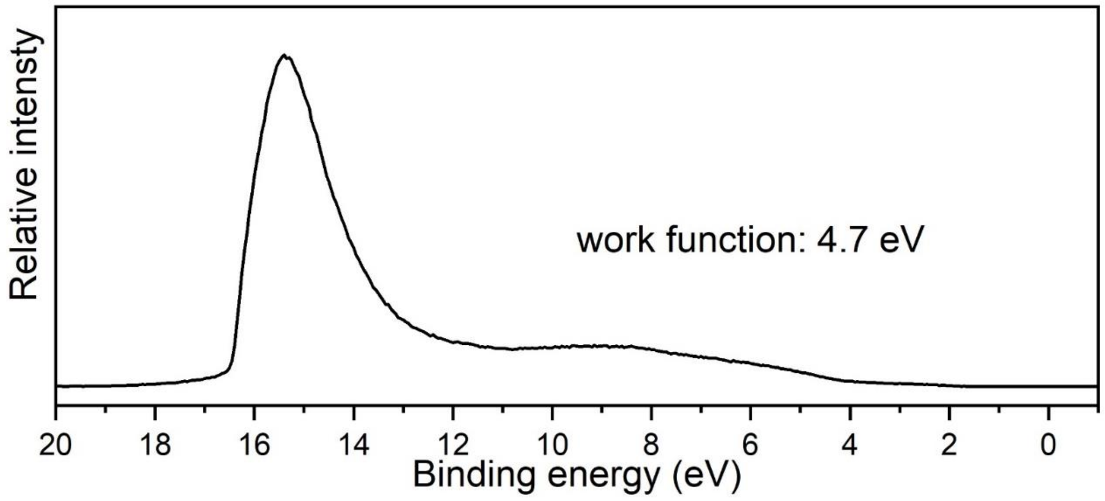

Figure S2.2 : UPS spectrum for the Carbon paste

step	Metals :	Au		Ag		Al	
		CPD(V)	delta	CPD(V)	delta	CPD(V)	delta
1	ambient	-0,490		-0,318		0,823	
2	7×10^{-5} mbar	-0,496	- 0,006	-0,305	0,013	0,867	0,044
3	1.2×10^{-5} mbar	-0,509	- 0,013	-0,303	0,002	0,889	0,022
4	ambient	-0,454	0,055	-0,331	-0,028	0,910	0,021

Table S2.1 : Kelvin probe measurements for the various bare metals, cycling between air and vacuum. The CPD values are defined as the work function of the tip minus the work function of the sample, i.e., more negative values correspond to a higher work function. Delta shows the difference between the values in each step.

The CPD (contact potential difference) measurements were performed using a McAllister KP6500 digital Kelvin probe, which features a stain-less steel tip wiat a 2 mm diameter as the reference electrode. The accuracy of the system is 1 mV. The Kelvin Probe Measurements were performed for each sample in the following sequence: under ambient conditions, under high vacuum at 7×10^{-5} mbar and at 1.2×10^{-5} mbar and finally again under ambient conditions.

S3: Time dependence of the open voltage under a temperature gradient contribution.

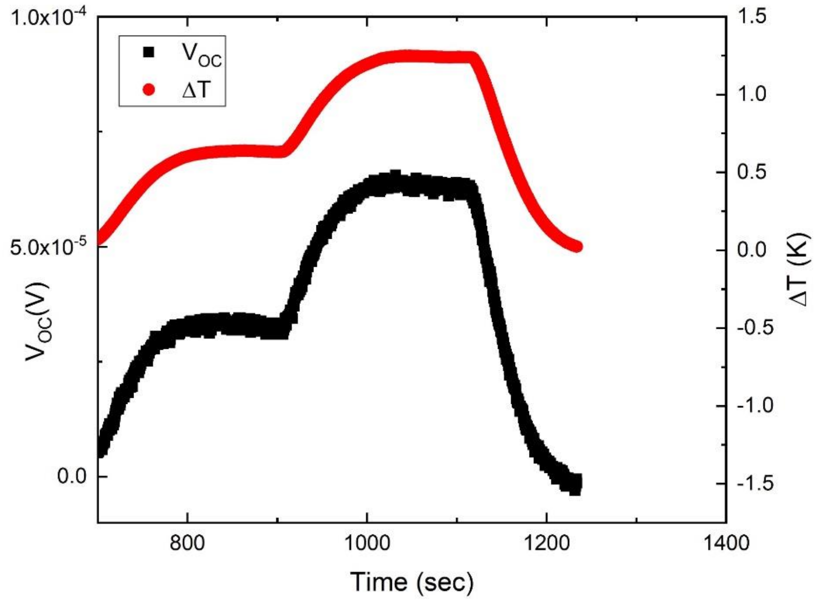

Figure S3.1: The open circuit voltage and the temperature difference as a function of time, as recorded for the PEDOT:Tos/Pt sample.

S4: IRAS spectra and analysis

For the IRAS samples, the PEDOT films were transferred on top of glass slides covered with the various metals (~100 nm thick) with dimensions of 25x10 mm^2. The IRAS spectra were acquired using a Bruker Equinox 55 FTIR spectrophotometer.

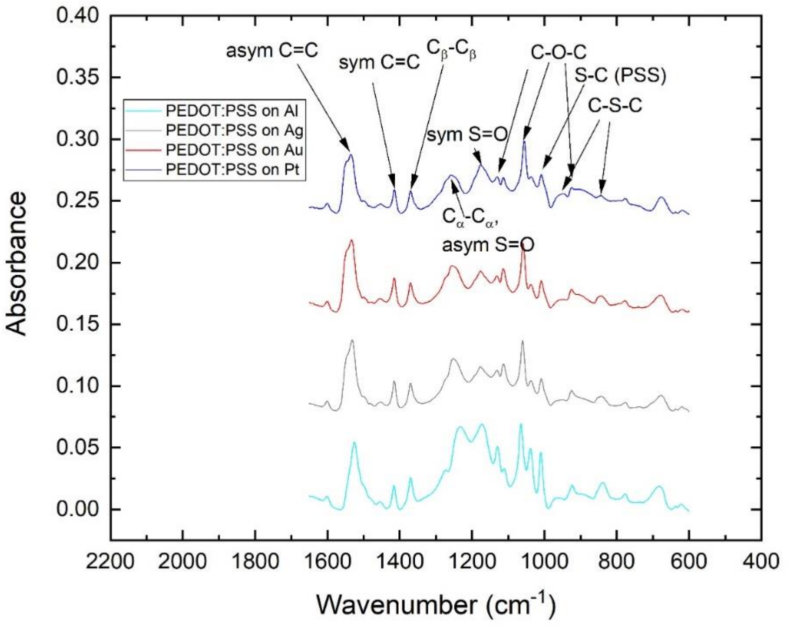

Figure S4.1: IRAS spectra of PEDOT:PSS of the same thickness on the various metals. The vibrational bands are depicted in the Figure and are also listed in Table S1.

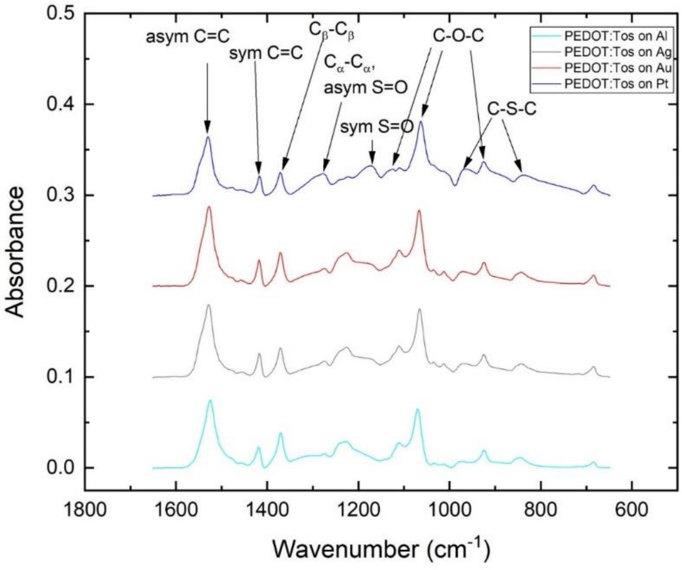

Figure S4.2: IRAS spectra of PEDOT:Tos films of the same thickness on the various metals. The vibrational bands are depicted in the Figure and are also listed in Table S1.

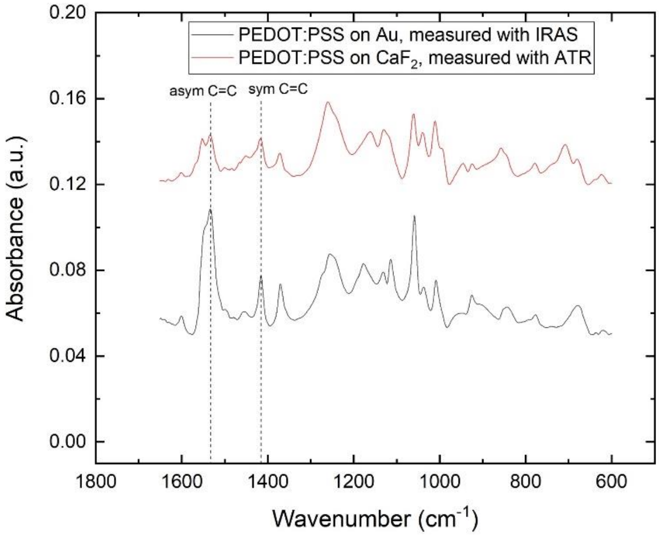

Figure S4.3 : The ATR-FTIR spectra for PEDOT:PSS on CaF_2 compared with the IRAS spectra of PEDOT:PSS/ Au.

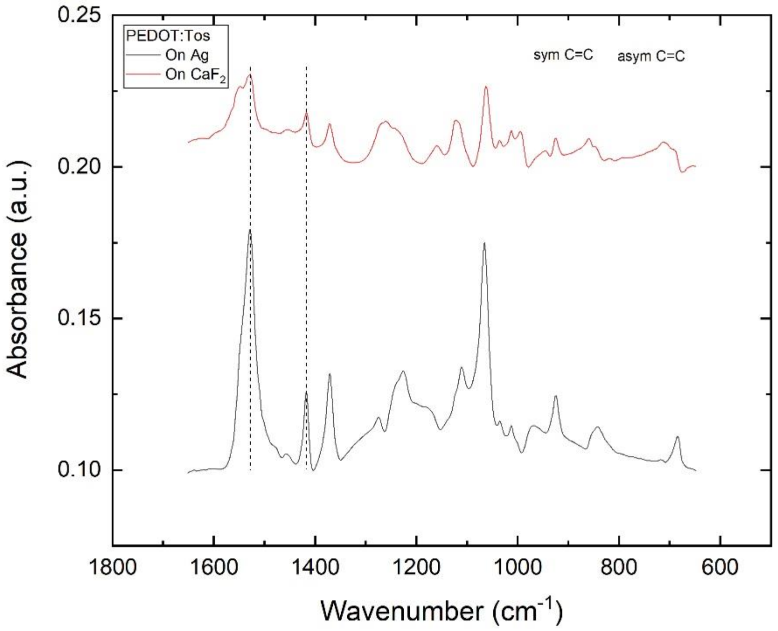

Figure S4.4 : The ATR-FTIR spectra for PEDOT:Tos on CaF_2 compared with the IRAS spectra of PEDOT:Tos/ Au.

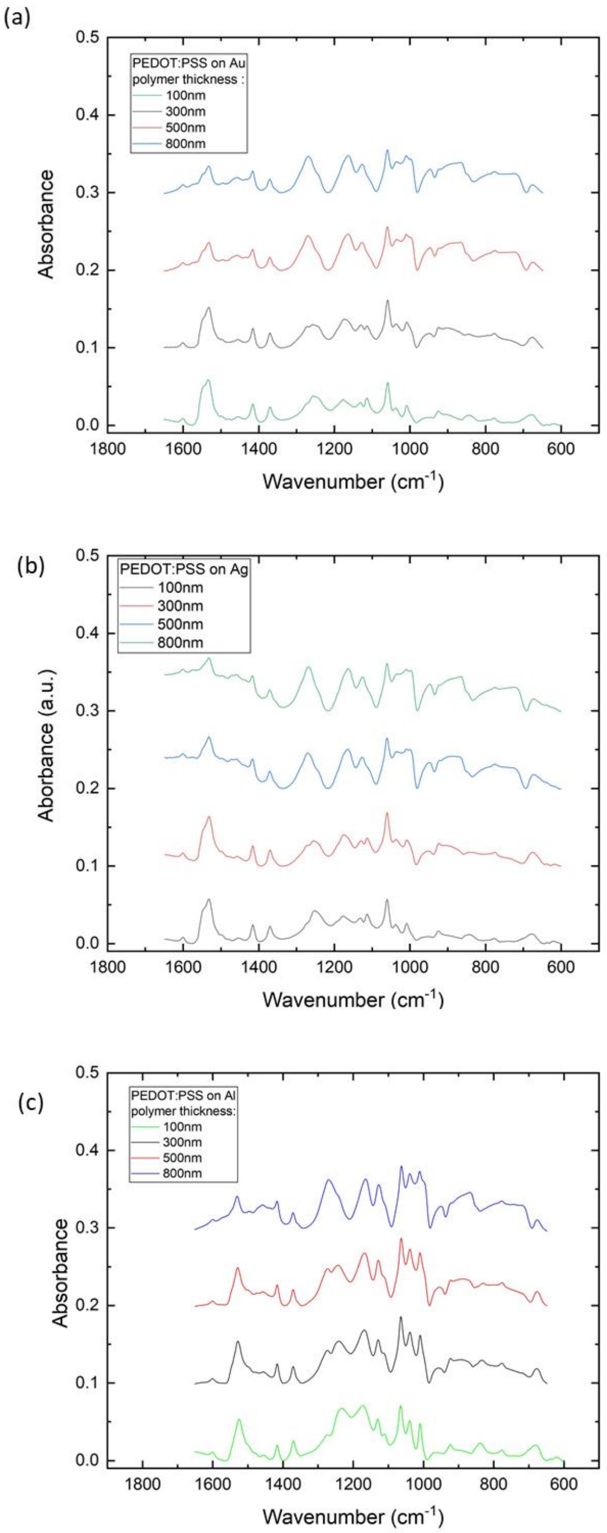

Figure S4.5: The IRAS spectra for different thickness PEDOT:PSS layers on (a) Au, (b) Ag, and (c) Al.

Table S4.1: FTIR peak maxima and chemical designations for PEDOT:PSS and PEDOT:Tos[19,20].

Peak maxima (cm^{-1})	PEDOT:PSS	PEDOT:Tos
1525-1535	Asymmetric C=C	Asymmetric C=C
1540-1550	Asymmetric C=C doublet	Asymmetric C=C doublet
1412-1418	Symmetric C=C	Symmetric C=C
1370	C_β- C_β	C_β- C_β
1273	C_α- C_α , asymmetric S=O	C_α- C_α , asymmetric S=O
1172	Symmetric S=O	Symmetric S=O
1126, 1055, 922	C-O-C	C-O-C
1026	S-C (PSS)	--
955, 842	C-S-C	C-S-C

S5 : Oxidation level estimate in the interfacial region and effect on contact resistance

We can trace back the oxidation level of PEDOT via the FTIR spectra recorded in **Fig. 3**. Indeed, the vibrational frequencies of the C=C asymmetric and C=C symmetric peaks are coupled to the oxidation level as pointed out in the paper by Khan *et al.* for PEDOT:Tos thin films.[19] Moreover, Khan *et al.* recorded both the normalized optical absorbances of PEDOT, the electrical conductivity, and the frequencies of the C=C asymmetric/symmetric peaks.[19] Hence, we were able to correlate our FTIR data with optical absorption. Combining these information together with the evolution of the normalized absorbance vs. oxidation levels for PEDOT:Tos provided by Bubnova *et al.*[6], we can estimate of the oxidation levels in the interfacial region probed by FTIR at the metal-PEDOT interface. We provide the details of this correlation in the **Table S5**.

We estimate that the PEDOT:Tos/Al has an oxidation level of ~17%. Moreover, our wavenumber shifts (**Fig.3**) for PEDOT:Tos/Al are similar to PEDOT:Tos that has been exposed to a pH ~ 10 solution, thus having a conductivity of ~250 S/cm (according to Khan *et al*).[19] For our films, this would correspond to a resistance about ~ 400 Ω. The OTEG single leg resistance metal/PEDOT/metal is constituted by the sum of contact resistances ($R_{con1;}$ R_{con2}) and the channel PEDOT resistance, $R_{dev} = R_{con1}+R_{channel}+R_{con2}$. The measured total resistance for Al/PEDOT:Tos/Al is R_{dev} ~ 900 Ω. If we consider that $R_{con1}=R_{con2}$= 400 Ω and $R_{channel}$ ~100 Ω , then find back the excepted measured R_{dev} = 900 Ω. This supports our train-of-thoughts about the oxidation level extraction that we conducted. Note however, the presence of very thin oxide layer (10-20 Å) on low work function metals (Ni, Ag, Al) could also contribute slightly to the contact resistance (max few Ohms), but the main resistance is expected to come from the oxidation level of PEDOT in the interfacial chemistry region. The presence of these thin natural oxide layers are detectable by impedance spectroscopy (**Section S7, Fig. S.7.3**).

pH	Symmetric cm-1	Asymmetric cm-1	Absorbance at 600nm	Metal	Symmetric on metal cm-1	Asymmetric on metal cm-1	Estimated absorbance @600nm	Oxidation level from Bubnova's paper
0	1417.406	1526.362	--	Al	1419	1523	~0.37	17%
1.1	1417.406	1525.9	0.32186	Ag	1417	1527	~0.35	19%
2.2	1417.406	1526.362	0.30784	Au	1417	1528		
3.17	1417.406	1528.29	0.30573	Pt	1415	1530		
4.17	1417.406	1528.29	0.3029					
5.5	1416.441	1529.255	0.31095					
6.2	1417.406	1528.29	--					
7	1416.441	1528.29	--					
8.16	1417.406	1529.255	0.35898					
9.12	1417.406	1529.255	--					
10.2	1417.406	1525.398	0.36998					
11.23	1420.298	1521.541	--					
12.5	1429.94	1518.648	0.37423					
13.2	1430.905	1518.648	--					

Table S5.1 : The wavenumber data from Khan *et al* [19](in red) and the comparison with our data and the extraction of the oxidation levels from the paper of Bubnova *et al* [6](in blue).

S6: Original GIWAXS patterns

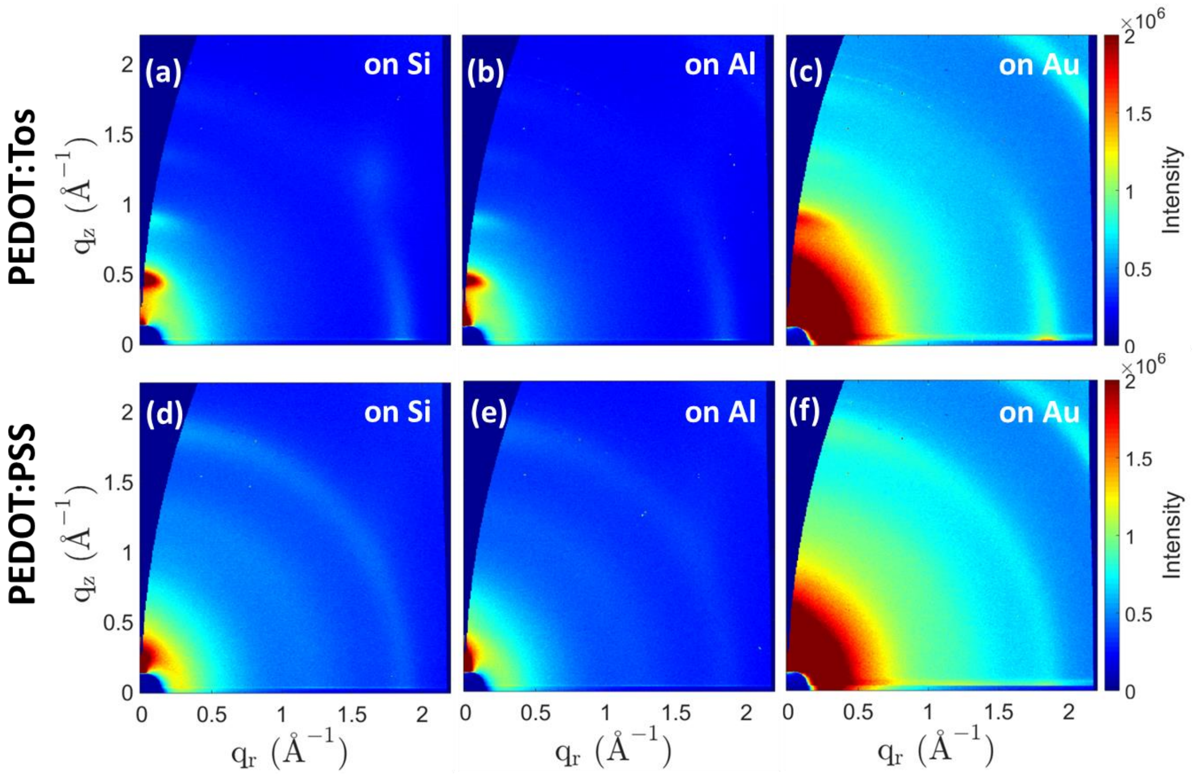

Figure S6.1 : The original 2D GIWAXS images for PEDOT:Tos (a)-(c) and PEDOT:PSS (d)-(f) on Si, Al, and Au before background subtraction.

Grazing incidence wide angle X-ray scattering (GIWAXS) experiments were performed at the NCD-SWEET beamline of the ALBA Synchrotron, located in Cerdanyola del Vallès, Spain. The wavelength of the X-rays was 0.9998 Å (12.4 keV), the sample to detector distance was 21.45 cm, and the angle of incidence was 0.16°. The diffracted intensity was recorded using a Rayonix LX255-HS detector, which consists of a pixel array of 960 × 2880 (H × V) and a pixel size of 88×88 μm^2. Data were normalized by the incident photon counts. Flat field, polarization, solid angle, and efficiency corrections were subsequently applied to the 2D GIWAXS images. The scattering vector q was defined with respect to the center of the incident beam and has a magnitude of $q=(4\pi/\lambda)\sin(\theta)$, where 2θ is the Bragg reflection angle. The wedge-shaped corrected images are presented herein, with q_r and q_z being the in-plane and near out-of-plane scattering vectors, respectively, defined as follows:

q_x=(2π/λ)(cos(2θ_f)cos(α_f)-cos(α_i)), q_y=(2π/λ)(sin(2θ_f)cos(α_f)), q_z = (2π/λ)(sin(α_f)+sin(α_i)),$q_r^2 = q_x^2 + q_y^2$. α_f is the exit angle in the vertical direction and $2\theta_f$ is the in-plane scattering angle[21]. Therefore, the scattering vector is calculated as follows, $q^2 = q_r^2 + q_z^2$. The 1D scattering patterns were obtained by performing a radial integration of the 2D images with respect to the incident beam.

S7 : Electrochemical Impedance Spectroscopy

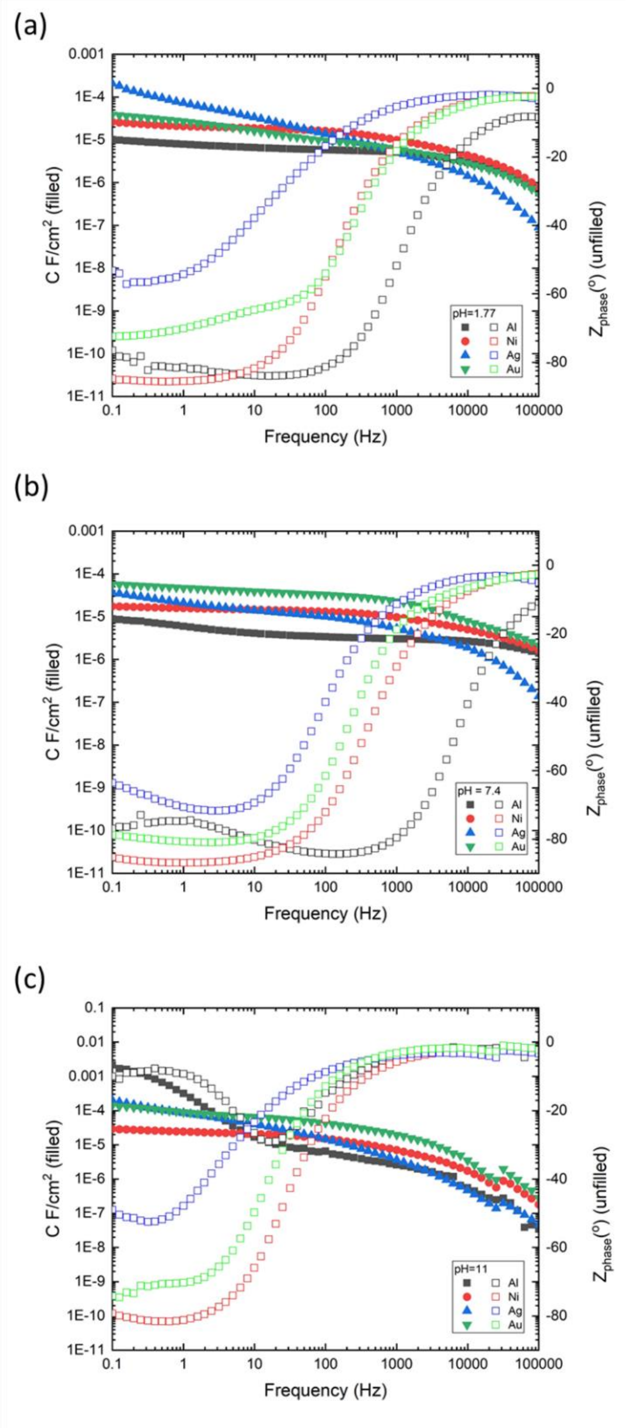

Figure S7.1 : The capacitance per area (as extracted from the Imaginary part of Impedance) and phase angle of the complex impedance for metal electrodes dip in electrolyte of pH=1.77, 7.4, 11.0.

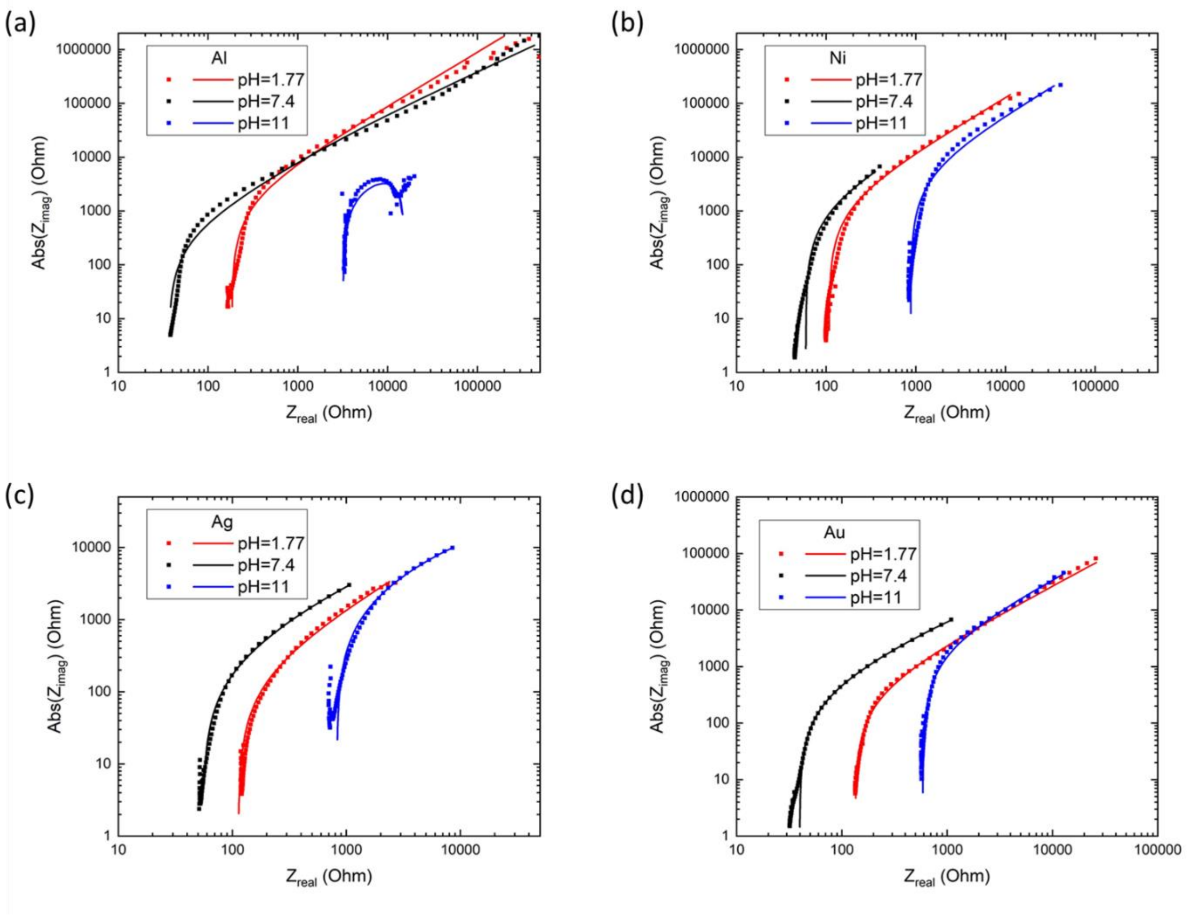

Figure S7.2 : The nyquist plots for the various metals in the various pH conditions. With a line are the fitted curves from the equivalent circuits (see **Table S7.1-3**)

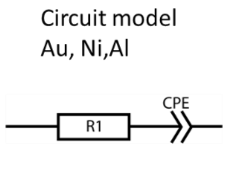

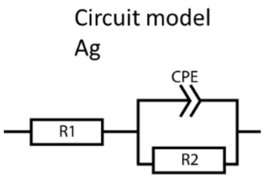


```
C = ((Q*R2)^(1/a))/R2
```

Circuit elements	R1 (Ohms)	R2 (Ohms)	Q / a	C (F)
Au	133.7	-	$1.9x10^{-5}$/0.77	-
Ni	107.2	-	$1.1x10^{-5}$/0.95	-
Al	183.5	-	$8.8x10^{-7}$/0.92	-
Ag	112.7	171568	$3x10^{-4}$/0.63	0.0033

Table S7.1 : The equivalent circuits and the respective parameters for the metals immersed in PSSH solution of pH=1.77

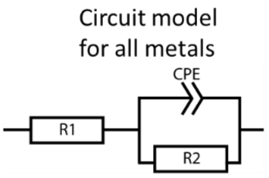

C = ((Q*R2)^(1/a))/R2

Circuit elements	R1 (Ohms)	R2 (Ohms)	Q/a	C (F)
Au	39.7	234572	$2.7x10^{-5}/0.91$	$3.2x10^{-5}$
Ni	59.1	24421820	$8.4x10^{-6}/0.96$	$1.0x10^{-5}$
Al	36.4	488879	$3.7x10^{-7}/0.92$	$8.3x10^{-7}$
Ag	55.4	42930	$6.5x10^{-5}/0.83$	$8.0x10^{-5}$

Table S7.2 : The equivalent circuits and the respective parameters for the metals immersed in PBS solution of pH=7.4

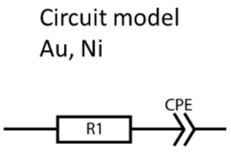

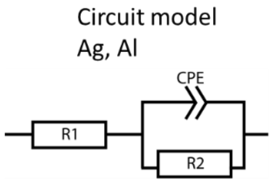

C = ((Q*R2)^(1/a))/R2

Circuit elements	R1 Ohms	R2 Ohms	Q farads / a	C farads
Au	587.1	-	$3.1x10^{-5}/0.82$	-
Ni	875.8	-	$7.1x10^{-6}/0.89$	-
Al	3190.2	11996	$1.2x10^{-5}/0.62$	$3.7x10^{-6}$
Ag	824.4	55258	$9.8x10^{-5}/0.7$	0.0002

Table S7.3 : The equivalent circuits and the respective parameters for the metals immersed in KOH solution of pH=11

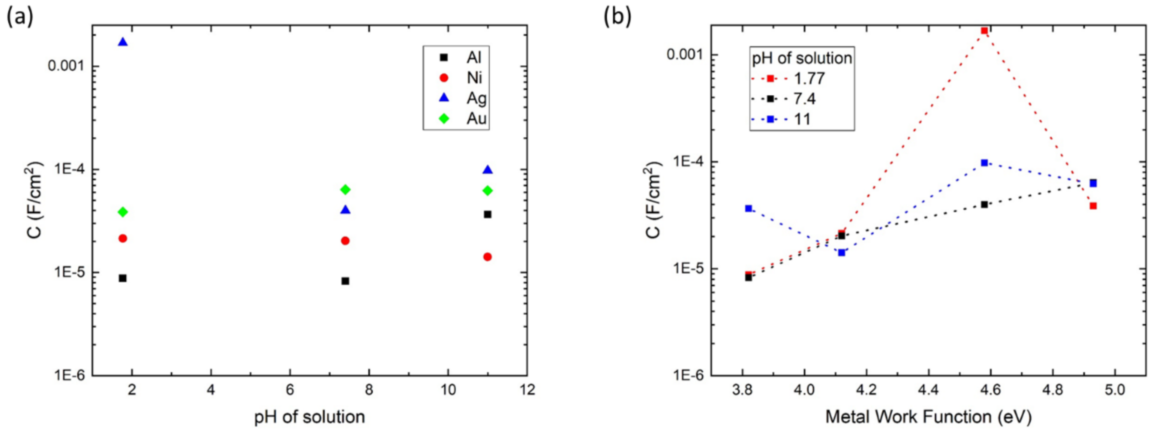

Figure S7.3 : The Electric Double Layer Capacitance per area as extracted from the fitted equivalent circuits for the various metals and conditions (a) vs. the pH of the exposed solution and (b) vs. the metal work function. Interrestingly, in the neutral pH solution, the native oxide on the surface of all metals considered is stable. For the same PBS electrolyte, the electric double layer capacitance is actually slightly different with the metals. This is illustrative to the presence of the thin oxide layer on certain metals and its absence for gold. The EDLC value for Au is then the largest and it decreases for lower workfunction electrode that have also a higher affinity for oxidation.

Electrochemical Impedance Spectroscopy was conducted in the four systems, Al, Ni, Ag, Au, in order to investigate the stability of the metals with respect to corrosive environments and extract the respective Electric Double Layer Capacitance (EDLC). In order to define the proper acidic pH, the pH of PEDOT:PSS dispersion was measured with a pH-meter, Thermo Scientific Orion Star A211 pH Benchtop Meter , and the respective pH was 1.77. Then, Poly(4-styrenesulfonic acid) solution was diluted so as to reach this pH value and it was used as the solution for the Electrochemical Impedance Spectroscopy. Additionally, PBS buffer solution (pH ~ 7.4) and KOH solution (1mM, pH = 11) were used as additional solution, so as to acquire a full image on the stability of the four metals. The metals were used as the working electrode in a three electrode configuration (Ag/AgCl reference and Pt mesh counter electrodes) and they were characterized with a Gamry Potentiostat. In **Fig. S7.1** and **Fig. S7.2** are presented the results of the spectra and in **Table S7.1-3** are the equivalent circuits and the

fitted parameters. As it can be observed in **Fig. S7.1** and **Fig. S7.2** all four metals are relatively stable in acidic and neutral conditions, while in basic conditions Al and Ag are starting to corrode in lower frequencies, which is evident from the respective increase of the phase (towards 0°) in those frequencies. For all metals, the EDLC was in the order of magnitude of 10^{-5}…10^{-4} F/cm^2 . EDLC is a means to characterize and understand the formation of interfacial layers between the metal and the media (i.e. the ions with water in this case). In order to acquire a more deep understanding on how this interfacial property is linked to the pH conditions and the metal work function, we plotted EDLC vs the pH and vs the metal work function (**Fig. S7.3**). Although for acidic or basic pH, there doesn't appear any correlation between the parameters, for neutral pH the EDLC is increasing with the metal work function; this is an indication that an oxide layer is formed between the metal and the electrolyte that decreases the capacitance of the system. Upon removal of that layer , i.e. in basic pH, EDLC is much higher.

S8 : Inductively Coupled Plasma with Optical Emission Spectroscopy

The metal samples (1x1 cm^2) were immersed in the different pH solutions (~5ml) for 30mins, to emulate the EIS in **Section S7**. Then those solution were dissolved to 10ml with additional DIW and a total of 16 samples were delivered to Gränges R&I for analysis with ICP-OES. The samples were in plastic tubes, approximately in volume of 10 ml of each. The analysis was done without any sample preparation, except addition of 0.5 ml of nitric acid on each. The samples and internal Wilab numbers are found in **Table S8.1**. The used wavelengths for analysis were:

Nickel 231.604 nm

Aluminium 167.078 nm

Silver 328.068 nm

Gold 242.795 nm

The instrument used for the analysis was Spectro Blue SOP inductive coupled plasma optical emission spectrometer (ICP-OES) and the results are presented as optical spectrum for sample intensities on analytical lines. The actual wavelength is marked as vertical line on spectrum and the results are presented in **Fig. S8.1-4**. As observed from Table S8.1 and Fig. S8.1-4, Ni and Au were relatively stable in the various pH solutions, while Al was unstable in the KOH and Ag was unstable in the PSSH and KOH solutions. Those findings are in agreement with the EIS in **Section S7**.

Table S8.1. Sample number, sample description, internal Wilab number and the approximate concentration of the analysed metal, the given pH accords to pH during the customer experiment, the addition of nitic acid made all samples acidic before ICP-OES analysis.

Sample number	Sample descripton	Internal Wilab-number	Approximate concentration (ppm)
1	Reference: pH=7.4 electrolyte PBS	B21-0276	-
2	Reference: DI water	B21-277	-
3	Reference: pH= 11 electrolyte KOH	B21-278	-
4	Reference: pH=1.8 electrolyte PSS	B21-279	-
	Nickel: solutions that has been in contact with a Ni surface with natural oxide		
5	Phosphate-buffered saline (pH=7.4)	B21-280	Ni ~ 0.03
6	KOH solution (1mM, pH = 11)	B21-281	Ni<0.01
7	polystyrene sulfonic acid (pH=1.8)	B21-282	Ni ~ 0.03
	Aluminum: solutions that has been in contact with a Al surface with natural oxide		
8	KOH solution (1mM, pH = 11)	B21-283	Al ~ 0.7
9	polystyrene sulfonic acid (pH=1.8)	B21-234	Al<0.1
10	Phosphate-buffered saline (pH=7.4)	B21-285	Al<0.1
	Silver: solutions that has been in contact with a Ag surface with natural oxide		
11	KOH solution (1mM, pH = 11)	B21-286	Ag~0.14
12	polystyrene sulfonic acid (pH=1.8)	B21-287	Ag~0.5
13	Phosphate-buffered saline (pH=7.4)	B21-288	Ag~0.03
	Gold: solutions that has been in contact with a Au surface with natural oxide		
14	polystyrene sulfonic acid (pH=1.8)	B21-289	-
15	Phosphate-buffered saline (pH=7.4)	B21-290	-
16	KOH solution (1mM, pH = 11)	B21-291	-

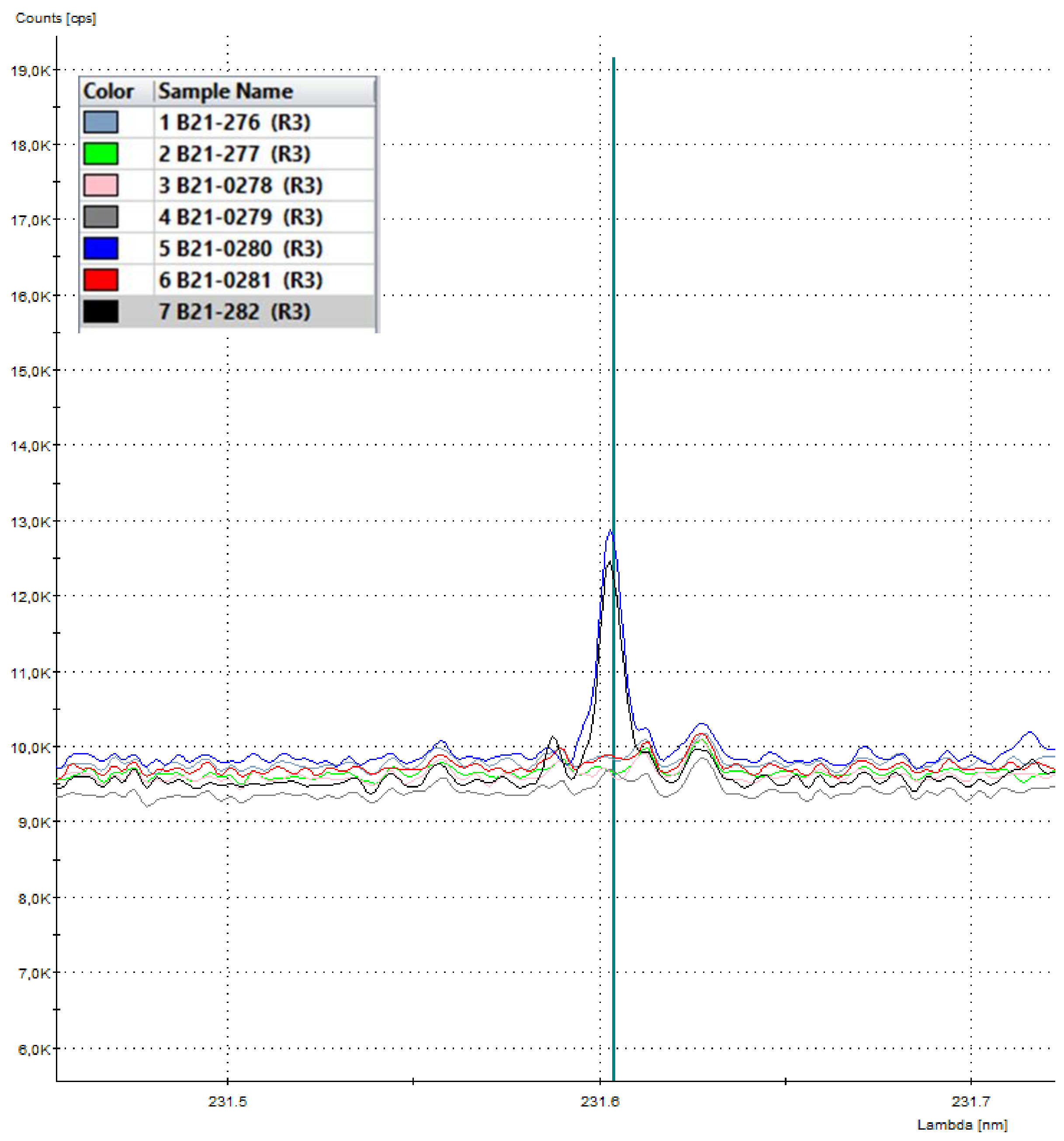

Figure S8.1: Analysis for Ni. Optical spectrum for the zero samples (sample 1-4) and solutions that has been in contact with a Nickel surface with natural oxide (samples 5-7)

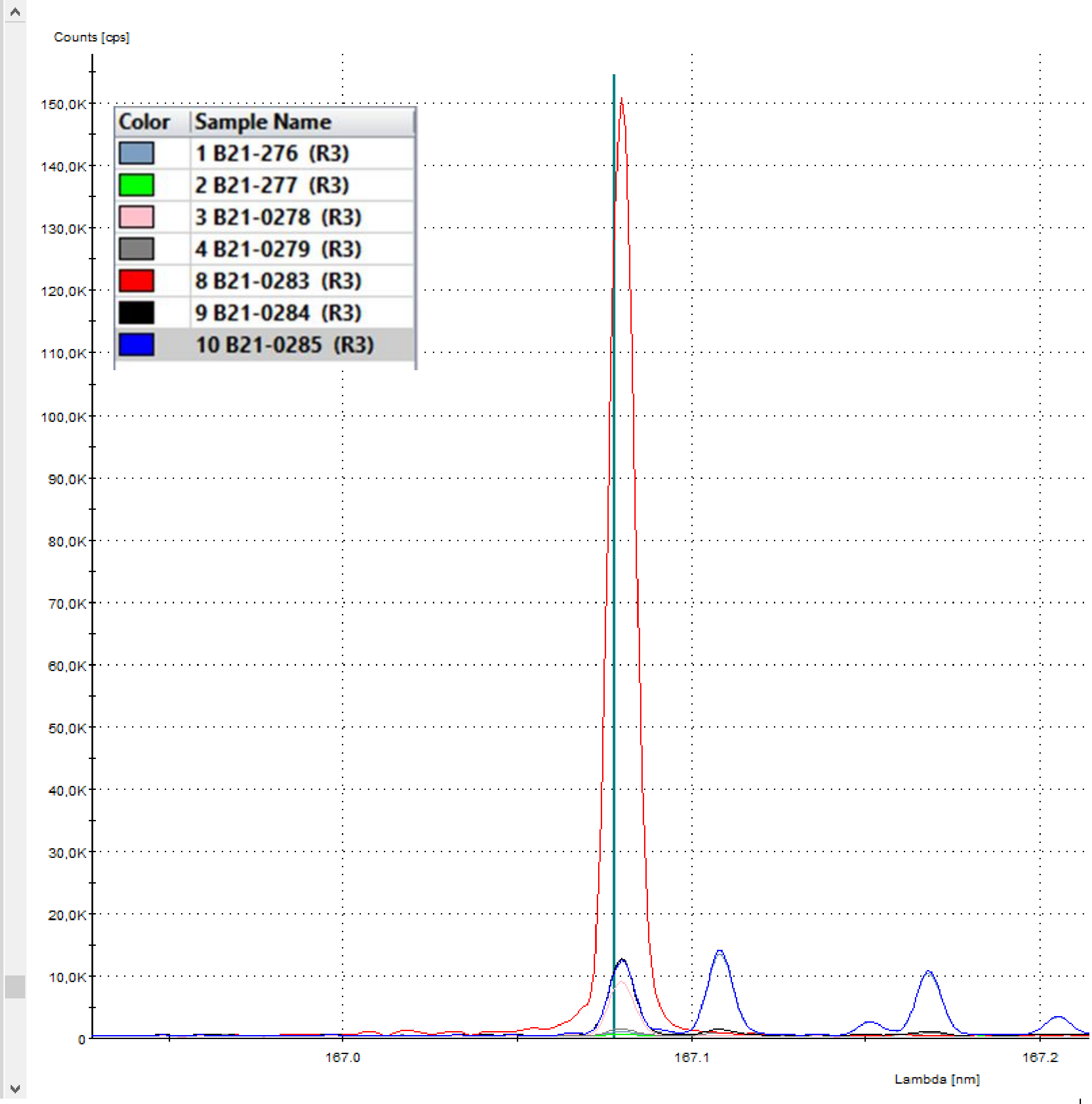

Figure S8.2 : Analysis for Al. Optical spectrum for the zero samples (sample 1-4) and solutions that has been in contact with a aluminum surface with natural oxide (samples 8-10)

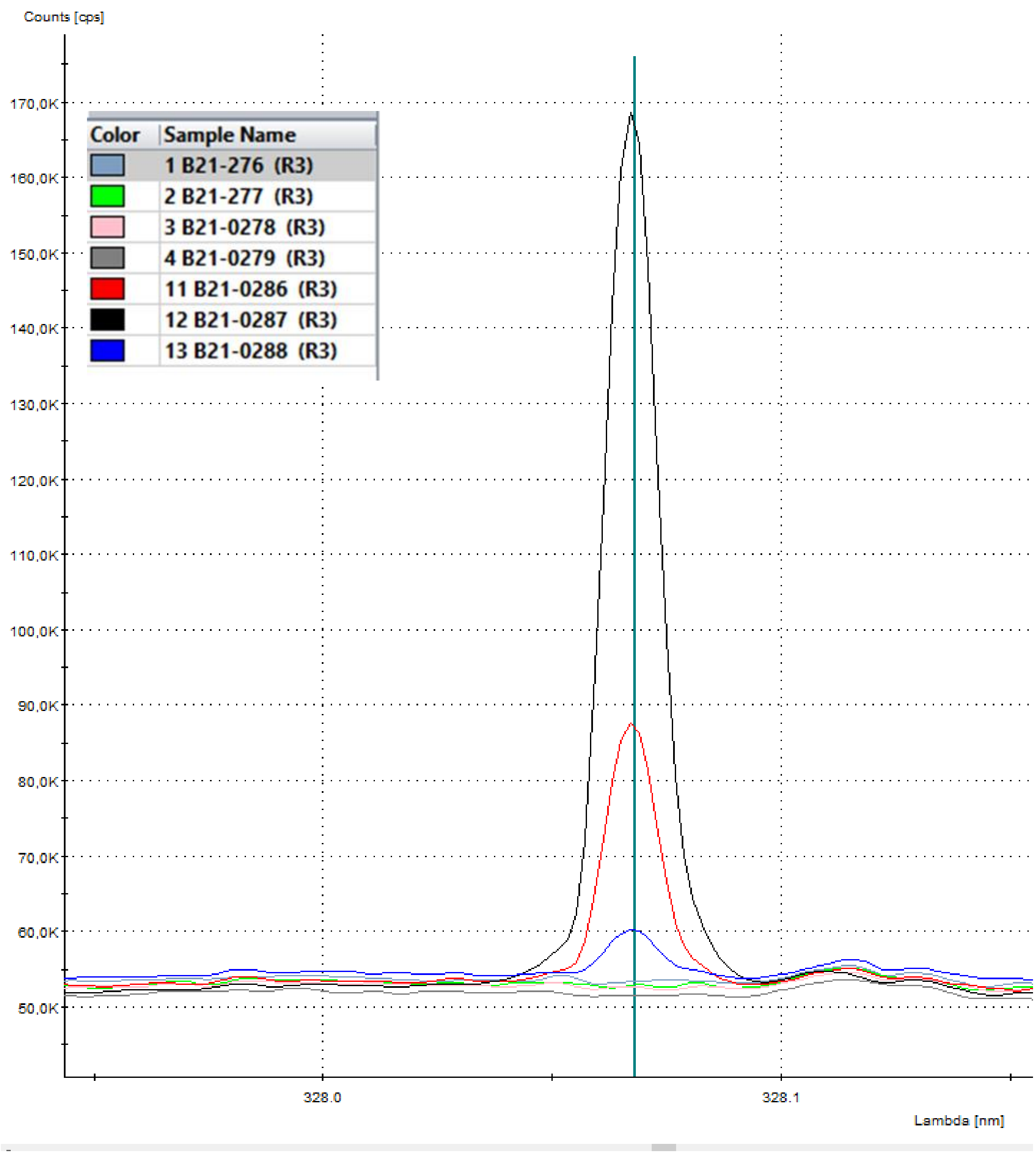

Figure S8.3: Analysis for Ag. Optical spectrum for the zero samples (sample 1-4) and solutions that has been in contact with silver surface with natural oxide (samples 11-13)

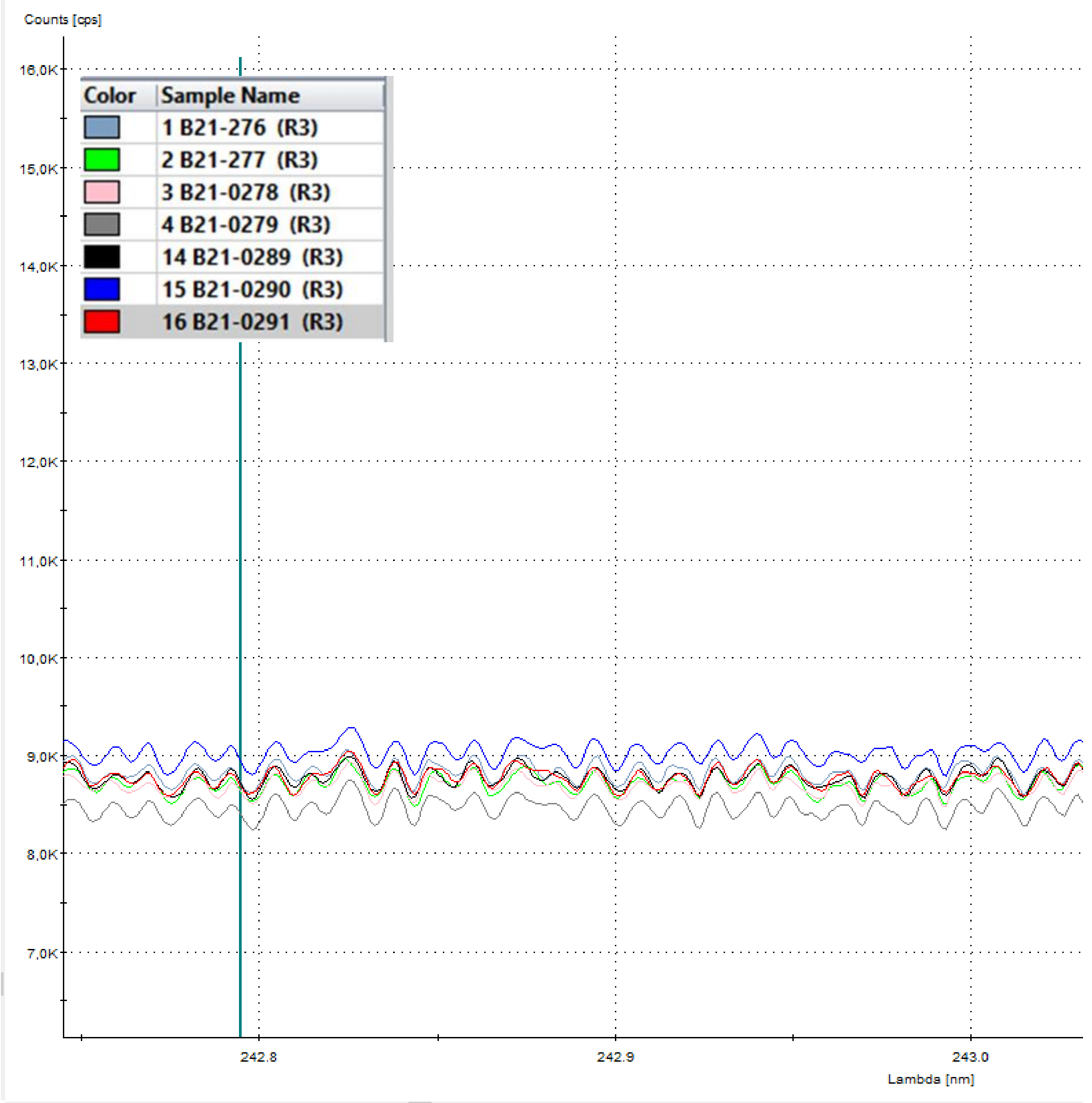

Figure S8.4: Analysis for Au. Optical spectrum for the zero samples (sample 1-4) and solutions that has been in contact with gold surface with natural oxide (samples 14-16)

S9 : Scanning Electron Microscopy

Scanning electron microscopy images (ZEISS Sigma 500 Gemini, 3kV) were recorded for all 4 metals , in pristine form and after exposure in the solutions of **Section S7** for 30 min (duration similar to the EIS experiments of **Section S7**). As it appears in **Fig. S9.1-4**, the surface microstructure of metals is basically unaffected by the exposure to the acidic and neutral solutions, while there appear to be several insulating islands at basic pH (evidence of corrosion). The SEM images are in agreement with the EIS observations of **Section S7**.

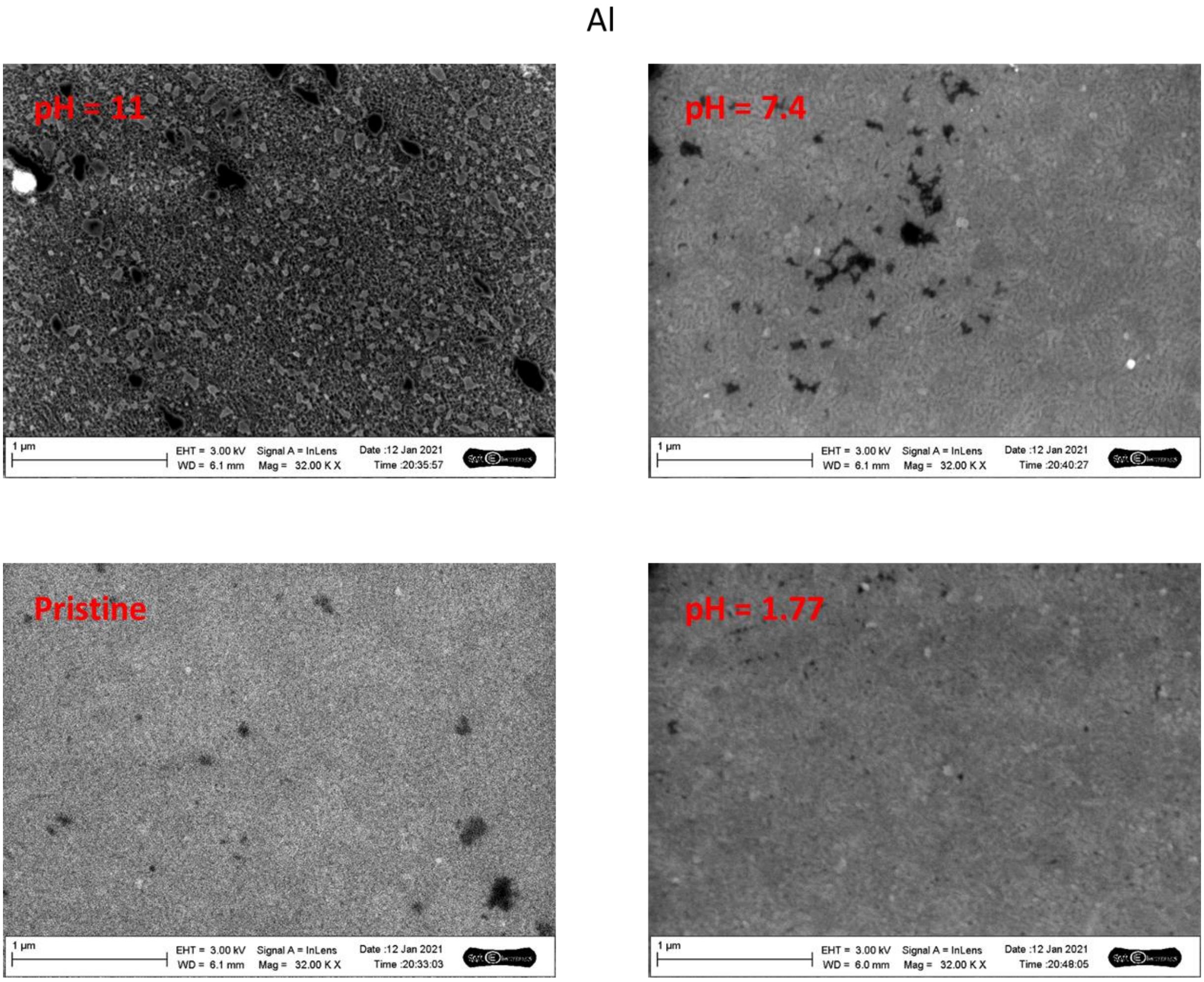

Figure S9.1 : The SEM images for Al; after exposure to solutions of pH=1.77, 7.4, 11, while pristine is an unexposed sample.

Ni

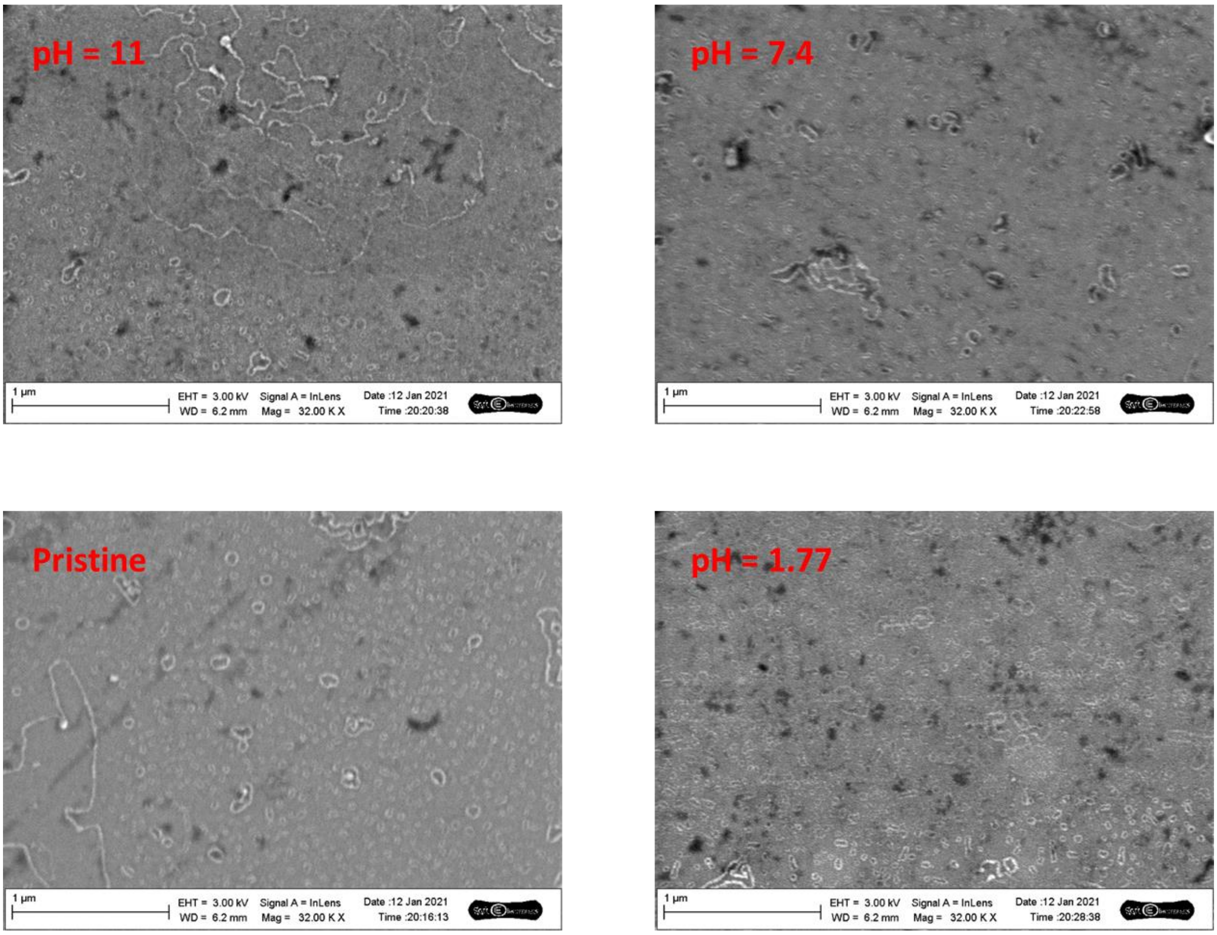

Figure S9.2 : The SEM images for Ni; after exposure to solutions of pH=1.77, 7.4, 11 , while pristine is un exposed sample.

Ag

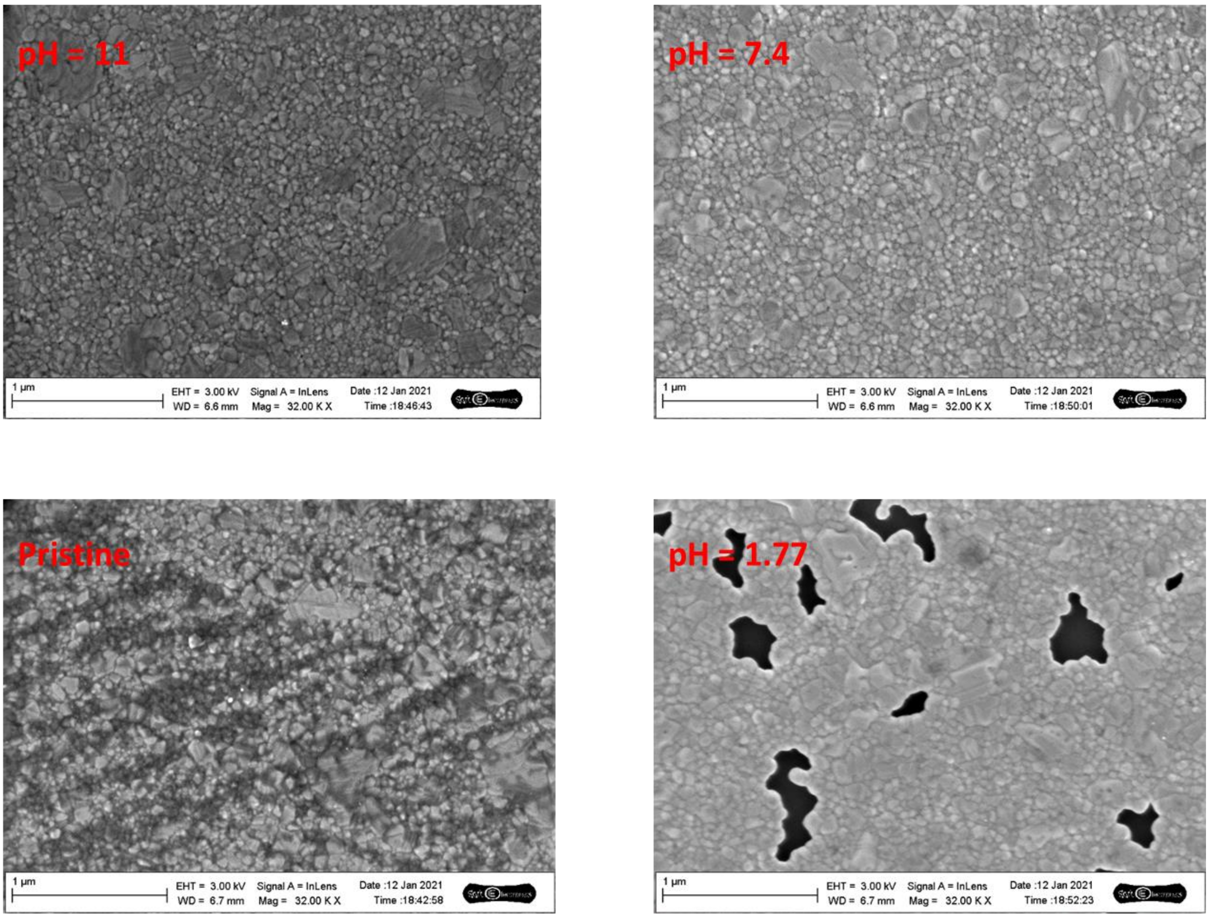

Figure S9.3 : The SEM images for Ag ; after exposure to solutions of pH=1.77, 7.4, 11 , while pristine is un exposed sample.

Au

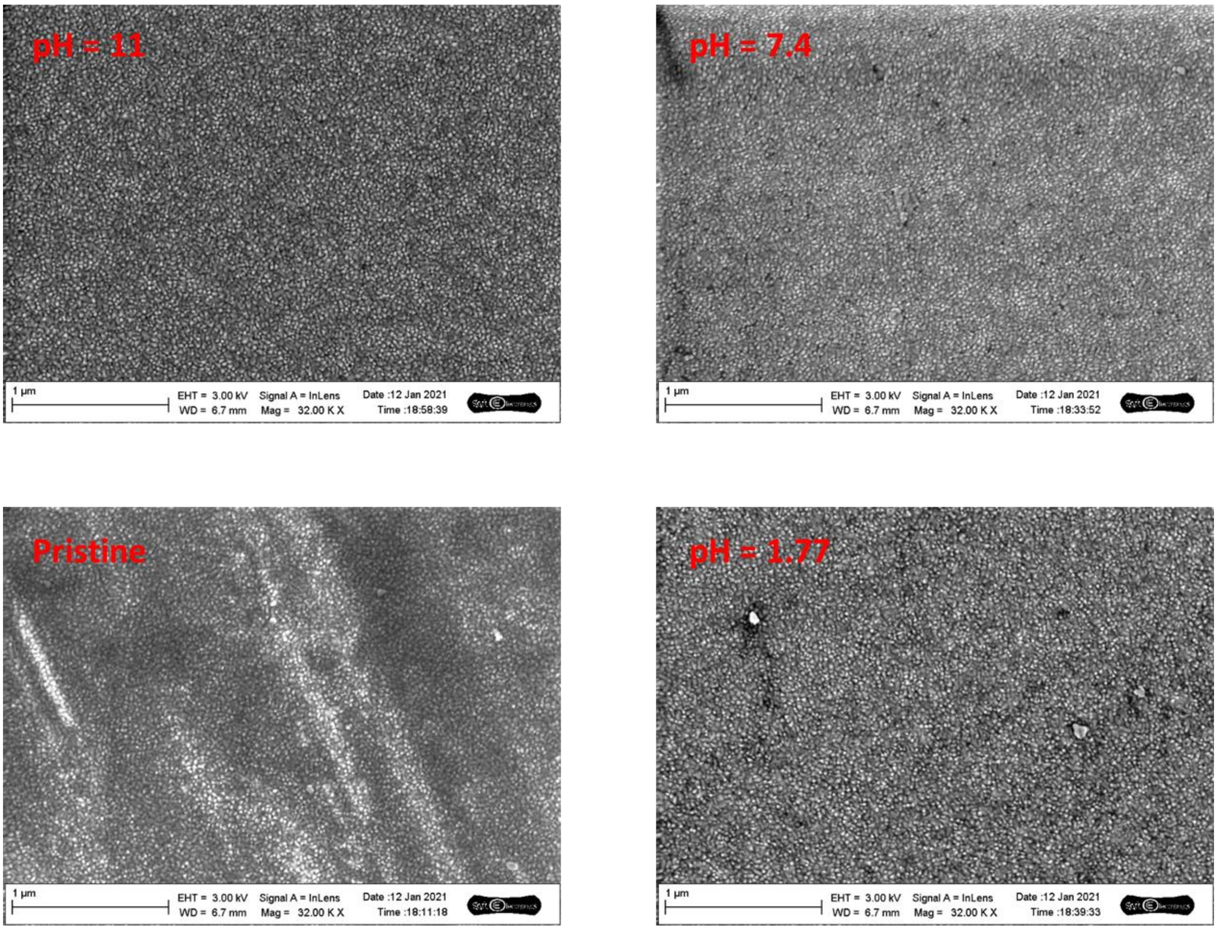

Figure S9.4 : The SEM images for Au ; after exposure to solutions of pH=1.77, 7.4, 11 , while pristine is un exposed sample.

S10: Dissimilar contact device measurement,

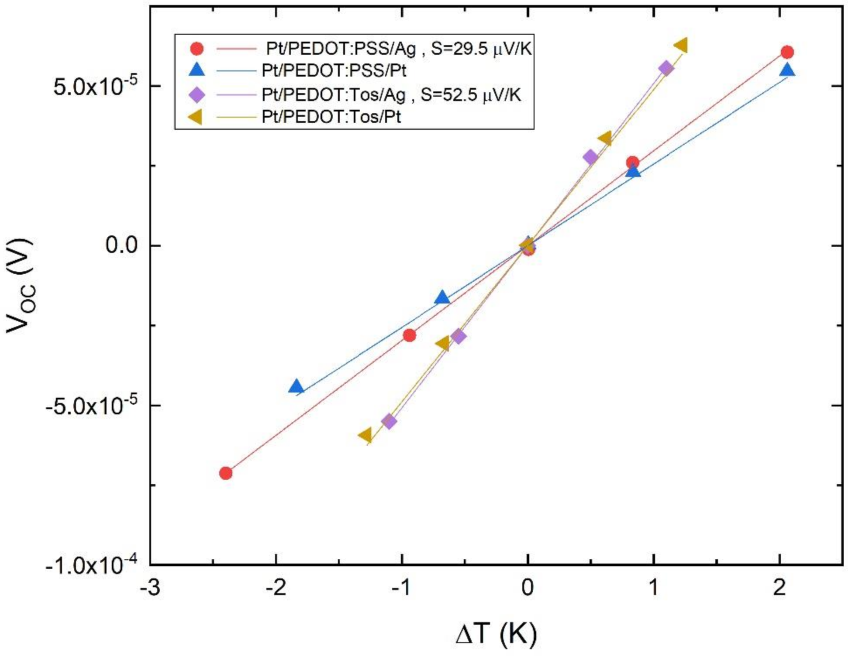

Figure S10.1 : The open circuit voltage (thermovoltage) plotted against the temperature difference for Pt/PEDOT:PSS/Ag, Pt/PEDOT:PSS/Pt, Pt/PEDOT:Tos/Ag, and Pt/PEDOT:Tos/Pt samples. In all cases R^2>0.99.

S11: Contact resistance device architecture

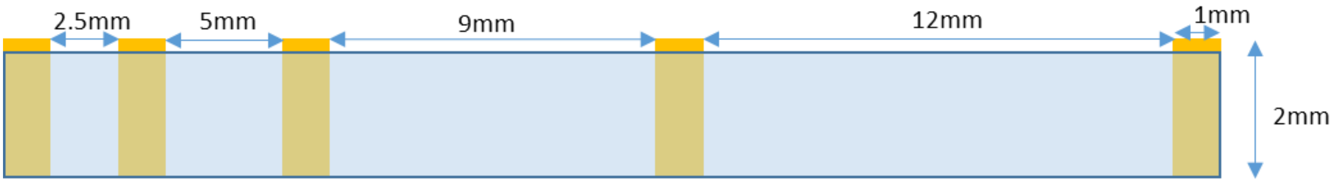

Figure S11.1 : The device architecture for the transmission line method. Yellow lines depict the electrodes and light-blue depicts PEDOT:PSS. For all systems the resistance of the single metal electrodes was 0.13 Ohm.

S12: Materials and Fabrication

3,4-ethylenedioxythiophene (EDOT), pyridine, dimethylsulfoxide (DMSO) , n-butanol, ethanol, acetone, isopropanol, diethyle ether, chlorobenzene, p-toluenesulfonic acid, sulfuric acid, and chloroplatinic acid were purchased from Sigma-Aldrich. Clevios CB54 and Clevios PH1000 were purchased from Heraus. Al, Ni, Cr, Ag, and Au were deposited with thermal evaporation on glass slides cleaned in an ultrasonic bath with acetone (5 min) and isopropanol (5 min). A ~5 nm layer of Cr was deposited prior to the deposition of Ag and Au for better adhesion to the glass slides. For all metals the deposition rates were 1 Å per second. Pt was electrodeposited on Au electrodes following the work of Strakosas *et al.*[22]. A solution containing 5 mM chloroplatinic acid and 50 mM sulfuric acid was used for the Pt electrodeposition in a three electrode planar configuration. Ag/AgCl and a platinum mesh were used as the reference and counter electrodes, respectively. A probe with a positioner was used to contact the Au electrode that was used as a working electrode for the electrodeposition. A potentiostat was used to apply -0.2V to the working electrode vs. the reference electrode for 5 minutes. The carbon paste DuPont 7102 was blade-coated on the samples.

P($g_4$2T-T) was synthesized with a procedure reported elsewhere[23]. 10mg/ml solutions of the copolymer in chlorobenzene were spincoated on the substrates (2000 rpm, 45 s, 1000 rpm/sec) and annealed at 120 ^{o}C for 30 mins. A solution of p-toluenesulfonic acid in diethyl ether (0.1M) was consequently spin coated on the samples to dope them. PEDOT:PSS dispersions were formulated by mixing Clevios PH1000 with 5 v-% DMSO[24], followed by spin coating (1500 rpm, 30 s, 800 rpm/sec) and annealing at 100 °C to remove the solvents. A part of the metal contact was then cleaned from PEDOT with a laboratory scrub that was dipped in distilled water. The samples were further annealed at 100 °C for 5 min. PEDOT:Tos was polymerized with in-situ wet chemical oxidative polymerization with a procedure that is reported elsewhere[3]. The Clevios CB54 was diluted at 40 wt-% in iron tosylate with n-butanol and then pyridine and DMSO were both added at 3 v-% concentration. The oxidant solution with the additives was left stirring for 12 hours and then stored in a fridge overnight before use. EDOT was added in a ratio of 3.3 μl per 100 μl of oxidant/additive solution. The EDOT/oxidant/additive was stirred vigorously for 30 s followed by spincoating on the desired substrate (1500 rpm, 30 s, 800 rpm/s). Afterwards the films/substrate were annealed at 100 °C for 15 min to initiate the polymerization. The films were washed for 5 min each in a bathing sequence of n-butanol, n-butanol, ethanol in order to remove excess oxidant. Finally, the samples were dried with nitrogen. As this polymerization is aggressive towards metals such as

Ag, the PEDOT:Tos samples were initially polymerized on top of a silicon wafer for all systems. During the washing treatment with n-butanol, the PEDOT films were delaminated with tweezers and transferred to the substrates with the metal contacts. Those samples were carefully dried with dry air. For the contact resistance measurements, two shadow masks were fabricated to extract the contact resistance with a 4-point probe approach and with the transmission line method. PEDOT:PSS was later deposited on those films similarly to the approach described earlier.

S13. Stability of metals at various pH

Now the stability of the metal oxide depends on the electrical potential and the pH of the environment as described by Pourbaix's diagram. We investigated the stability of our metal surface in electrolyte solutions of pH=1.8, 7.4 and 11 by Electrochemical Impedance Spectroscopy (EIS, see **Fig S7.1-3**) and Inductively Coupled Plasma with Optical Emission Spectroscopy (ICP-OES, see **Fig. S8.1-4**). The electrodes were dipped in the three different solutions and EIS was performed. All metal surfaces display a capacitive behaviour at low frequencies (phase angle approaches -90 deg in the range [0.1-10 Hz]) related to the formation of an Electric Double Layer Capacitance (EDLC) at the metal(oxide)/electrolyte interface of the order of [10^{-5}-10^{-4} µF/cm^2]. The close value of the EDLC (in neutral pH) for Ag, Ni, Al to the value of Au without oxide indicates again that those native oxide layers are very thin (**Section S7, Fig. S.7.3**). The values of the surface capacitance for Au are quasi-constant versus pH (~4×10^{-5} µF/cm^2, see **Fig. S7.3)**, while they are slightly lower for nickel because of the thin and semiconducting surface oxide (~1×10^{-5} µF/cm^2). The ICP-OES analysis does not reveal much dissolution of the metal oxide in the three different pH solutions for Ni and Au (**Fig. S8.1, Fig. S8.4**). In the **Fig S7.1**, only Ag in the three different pH displays some deviation with a slight resistive behavior at low frequencies, while Al clearly undergoes an electro-induced dissolution only at basic pH (phase angle is close to zero deg). The capacitance for Ag is highest for the acidic and basic pH; while for Al, it is highest for the basic pH. The high capacitance is attributed to the dissolution of the oxide layer, which is also confirmed by the presence of Al and Ag ions in those solutions after dipping the electrodes for ~30 mins as measured by ICP-OES analysis (**Fig. S8.2-3**). The observations are also in good agreement with the microstructure of the metal surface observed by Scanning Electron Microscopy (SEM, see **Section S9**) after the 30 min exposure in those electrolytes. It is only the microstructure of the Ag surface that is modified in basic and acidic medium, together with that of the Al surface in alkali medium.

References

1 Reenen, S. v. & Kemerink, M. Correcting for contact geometry in Seebeck coefficient measurements of thin film devices. *Organic Electronics* **15**, 2250-2255, doi:https://doi.org/10.1016/j.orgel.2014.06.018 (2014).

2 Petsagkourakis, I. *et al.* Structurally-driven Enhancement of Thermoelectric Properties within Poly(3,4-ethylenedioxythiophene) thin Films. *Scientific Reports* **6**, doi:10.1038/srep30501 (2016).

3 Petsagkourakis, I. *et al.* Correlating the Seebeck coefficient of thermoelectric polymer thin films to their charge transport mechanism. *Organic Electronics* **52**, 335-341, doi:https://doi.org/10.1016/j.orgel.2017.11.018 (2018).

4 Søndergaard, R. R., Hösel, M., Espinosa, N., Jørgensen, M. & Krebs, F. C. Practical evaluation of organic polymer thermoelectrics by large-area R2R processing on flexible substrates. *Energy Science & Engineering* **1**, 81-88, doi:https://doi.org/10.1002/ese3.8 (2013).

5 Li, Z. *et al.* A Free-Standing High-Output Power Density Thermoelectric Device Based on Structure-Ordered PEDOT:PSS. *Advanced Electronic Materials* **4**, doi:10.1002/aelm.201700496 (2018).

6 Bubnova, O. *et al.* Optimization of the thermoelectric figure of merit in the conducting polymer poly(3,4-ethylenedioxythiophene). *Nat Mater* **10**, 429-433, doi:10.1038/nmat3012 (2011).

7 Sun, Y. *et al.* Organic thermoelectric materials and devices based on p- and n-type poly(metal 1,1,2,2-ethenetetrathiolate)s. *Adv Mater* **24**, 932-937, doi:10.1002/adma.201104305 (2012).

8 Anno, H., Nishinaka, T., Hokazono, M., Oshima, N. & Toshima, N. Thermoelectric Power-Generation Characteristics of PEDOT:PSS Thin-Film Devices with Different Thicknesses on Polyimide Substrates. *Journal of Electronic Materials* **44**, 2105-2112, doi:10.1007/s11664-015-3668-x (2015).

9 Du, Y. *et al.* Thermoelectric fabrics: toward power generating clothing. *Sci Rep* **5**, 6411, doi:10.1038/srep06411 (2015).

10 Aranguren, P. *et al.* Optimized design for flexible polymer thermoelectric generators. *Applied Thermal Engineering* **102**, 402-411, doi:https://doi.org/10.1016/j.applthermaleng.2016.03.037 (2016).

11 Lee, W. *et al.* Acidity-Controlled Conducting Polymer Films for Organic Thermoelectric Devices with Horizontal and Vertical Architectures. *Scientific Reports* **6**, 33795, doi:10.1038/srep33795 (2016).

12 Ail, U. *et al.* Room temperature synthesis of transition metal silicide-conducting polymer micro-composites for thermoelectric applications. *Synthetic Metals* **225**, 55-63, doi:https://doi.org/10.1016/j.synthmet.2017.01.007 (2017).

13 Mukaida, M., Wei, Q. & Ishida, T. Polymer thermoelectric devices prepared by thermal lamination. *Synthetic Metals* **225**, 64-69, doi:https://doi.org/10.1016/j.synthmet.2016.11.016 (2017).

14 Taroni, P. J. *et al.* Toward Stretchable Self-Powered Sensors Based on the Thermoelectric Response of PEDOT:PSS/Polyurethane Blends. *Advanced Functional Materials* **28**, 1704285, doi:10.1002/adfm.201704285 (2018).

15 Kee, S., Haque, M. A., Corzo, D., Alshareef, H. N. & Baran, D. Self - Healing and Stretchable 3D - Printed Organic Thermoelectrics. *Advanced Functional Materials* **29**, doi:10.1002/adfm.201905426 (2019).

16 Mukaida, M., Kirihara, K. & Wei, Q. Enhanced Power Output in Polymer Thermoelectric Devices through Thermal and Electrical Impedance Matching. *ACS Applied Energy Materials* **2**, 6973-6978, doi:10.1021/acsaem.9b01342 (2019).

17 Ni, D., Song, H., Chen, Y. & Cai, K. Free-standing highly conducting PEDOT films for flexible thermoelectric generator. *Energy* **170**, 53-61, doi:https://doi.org/10.1016/j.energy.2018.12.124 (2019).

18 Kim, N. L., Samuel; Petsagkourakis, Ioannis; Mengistie, Desalegn Alemu; Kee, Seyoung; Ederth, Thomas; Gueskine, Viktor; Leclère, Philippe; Lazzaroni, Roberto; Crispin, Xavier; Tybrandt, Klas; . Elastic Conducting Polymer Composites in Thermoelectric Modules. *Nature Communications* (2020).

19 Khan, Z. U. *et al.* Acido-basic control of the thermoelectric properties of poly(3,4-ethylenedioxythiophene)tosylate (PEDOT-Tos) thin films. *J Mater Chem C Mater* **3**, 10616-10623, doi:10.1039/c5tc01952d (2015).

20 Mitraka, E. *et al.* Oxygen-induced doping on reduced PEDOT. *J Mater Chem A Mater* **5**, 4404-4412, doi:10.1039/c6ta10521a (2017).

21 Renaud, G., Lazzari, R. & Leroy, F. Probing surface and interface morphology with Grazing Incidence Small Angle X-Ray Scattering. *Surface Science Reports* **64**, 255-380, doi:https://doi.org/10.1016/j.surfrep.2009.07.002 (2009).

22 Strakosas, X. *et al.* Catalytically enhanced organic transistors for in vitro toxicology monitoring through hydrogel entrapment of enzymes. *Journal of Applied Polymer Science* **134**, doi:10.1002/app.44483 (2017).

23 Xu, K. *et al.* Ground-state electron transfer in all-polymer donor–acceptor heterojunctions. *Nature Materials* **19**, 738-744, doi:10.1038/s41563-020-0618-7 (2020).

24 Kim, G. H., Shao, L., Zhang, K. & Pipe, K. P. Engineered doping of organic semiconductors for enhanced thermoelectric efficiency. *Nat Mater* **12**, 719-723, doi:10.1038/nmat3635 (2013).
